# Generation of Individual Whole-Brain Atlases With Resting-State fMRI Data Using Simultaneous Graph Computation and Parcellation

**DOI:** 10.3389/fnhum.2018.00166

**Published:** 2018-05-04

**Authors:** J. Wang, Z. Hao, H. Wang

**Affiliations:** ^1^School of Mathematics and Big Data, Foshan University, Foshan, China; ^2^Key Laboratory of Child Development and Learning Science of Ministry of Education, Research Center for Learning Science, Southeast University, Nanjing, China

**Keywords:** whole-brain parcellation, resting-state fMRI, supervoxel, graph-without-cut, random parcellation

## Abstract

The human brain can be characterized as functional networks. Therefore, it is important to subdivide the brain appropriately in order to construct reliable networks. Resting-state functional connectivity-based parcellation is a commonly used technique to fulfill this goal. Here we propose a novel individual subject-level parcellation approach based on whole-brain resting-state functional magnetic resonance imaging (fMRI) data. We first used a supervoxel method known as simple linear iterative clustering directly on resting-state fMRI time series to generate supervoxels, and then combined similar supervoxels to generate clusters using a clustering method known as graph-without-cut (GWC). The GWC approach incorporates spatial information and multiple features of the supervoxels by energy minimization, simultaneously yielding an optimal graph and brain parcellation. Meanwhile, it theoretically guarantees that the actual cluster number is exactly equal to the initialized cluster number. By comparing the results of the GWC approach and those of the random GWC approach, we demonstrated that GWC does not rely heavily on spatial structures, thus avoiding the challenges encountered in some previous whole-brain parcellation approaches. In addition, by comparing the GWC approach to two competing approaches, we showed that GWC achieved better parcellation performances in terms of different evaluation metrics. The proposed approach can be used to generate individualized brain atlases for applications related to cognition, development, aging, disease, personalized medicine, etc. The major source codes of this study have been made publicly available at https://github.com/yuzhounh/GWC.

## Introduction

Since the first manifestation that specific brain areas are functionally connected in resting brain (Biswal et al., 1995), neuroscientists have been characterizing the human brain as networks (Sporns et al., 2005; Bullmore and Sporns, 2009). To construct brain networks, a critical step is to parcellate the brain into a specific number of functional units (Wig et al., 2011). However, no agreement has been reached on how the brain should be parcellated (Hallquist and Hillary, 2018).

Brain atlases generated based on meta-analysis, random criteria, structural criteria, and functional connectivity (Wig et al., 2011; de Reus and Van den Heuvel, 2013; Fornito et al., 2013; Stanley et al., 2013) have long been used to construct functional networks. Among them, resting-state functional connectivity (RSFC)-based parcellations (Craddock et al., 2012; Shen et al., 2013) draw significant attention because they are originally designed for such purpose and are more reliable. The common idea is to parcellate the brain into spatially contiguous, functionally homogeneous, and reproducible clusters. Many clustering algorithms have been applied in RSFC-based parcellations, e.g., spectral clustering (van den Heuvel et al., 2008; Craddock et al., 2012; Shen et al., 2013), K-means (Kim et al., 2010; Kahnt et al., 2012), and hierarchical clustering (Blumensath et al., 2013; Thirion et al., 2014). Some of these studies (Kim et al., 2010; Kahnt et al., 2012) focused on subdividing a region of interest (ROI) while the other studies focused on parcellating the whole brain. In this study, we introduce a novel RSFC-based whole-brain parcellation approach with the aim to improve the current parcellations.

Our study focused on individual subject-level parcellation rather than group-level parcellation. Previous studies have observed anatomical and functional variability across individuals (Mueller et al., 2013; Laumann et al., 2015). Individual subject-level parcellation captures this variability and leads to more reliable delineation of parcels (Wang D. H. et al., 2015; Chong et al., 2017; Tong et al., 2017). Therefore, it has recently become a mainstream in brain parcellation.

Most previous studies (Craddock et al., 2012; Shen et al., 2013; Thirion et al., 2014) generated clusters from voxels directly. In contrast, we used supervoxels as the building blocks of the clusters in this study. Specifically, we first aggregated similar voxels to generate supervoxels, and then combined similar supervoxels to generate clusters. Supervoxel methods (Veksler et al., 2010; Lucchi et al., 2012; Xu and Corso, 2012; Papon et al., 2013) effectively extract image structure, reduce image redundancy, provide a solid basis to compute local image features, and facilitate subsequent processing. Therefore, they are suitable for application in brain parcellation. The supervoxel method utilized in this study was simple linear iterative clustering (SLIC) (Lucchi et al., 2012). SLIC has been demonstrated to be superior to many existing superpixel algorithms in two-dimensional (2D) image segmentation tasks (Achanta et al., 2012). It has also been widely applied in three-dimensional (3D) image segmentation tasks (Lucchi et al., 2012; Menze et al., 2015). We have previously used SLIC to generate brain atlases (Wang and Wang, 2016; Wang et al., 2016). Both previous studies treated supervoxels as clusters in brain atlases. However, the generation of superpixels or supervoxels is commonly used as a pre-processing step in segmentation algorithms. Therefore, we combined similar supervoxels to generate clusters in this study.

To combine supervoxels, we utilized a state-of-the-art image segmentation approach known as graph-without-cut (GWC) (Gao et al., 2016). GWC is a graph-based approach, stemming from clustering with adaptive neighbors (CAN) (Nie et al., 2014). The CAN approach was originally designed to partition low dimensional data while the GWC approach was originally designed to segment 2D images. Traditional graph-based approaches organize the elements of an image into a graph and then partition the image based on the graph. GWC merges the two steps, i.e., calculating the graph and partitioning the image, into a single optimization problem. This algorithm design generates the optimal graph for segmentation. Both spatial information and multiple visual features of the image are considered in GWC. Additionally, GWC restricts the number of connected components in the obtained graph so that it is exactly equal to the initialized cluster number. Gao et al. (2016) have reported that GWC achieves better clustering performances than some existing image segmentation approaches. Therefore, we extended GWC to 3D space and applied it to perform whole-brain parcellation for individuals in this study.

After generating a brain atlas, it is important to ensure that the brain atlas does not rely heavily on spatial structures. Different parcellation approaches incorporate spatial structures in different ways. In the normalized cuts (Ncut) approach (Craddock et al., 2012), spatial structure is introduced by the spatial constraint in weight definition. In the SLIC approach (Wang and Wang, 2016), spatial structures are introduced by initializing an ideal geometric pattern, integrating the spatial distance into the unified distance, and searching in a local space. As Wang and Wang (2016) have shown, incorporating suitable spatial structures in whole-brain parcellation approaches is quite necessary to guarantee the spatial contiguity of the resultant clusters. However, parcellation approaches with excessive spatial structures would encounter three major problems (Craddock et al., 2012; Blumensath et al., 2013; Shen et al., 2013; Gordon et al., 2016; Wang and Wang, 2016). First, they tend to generate clusters with comparable shapes and sizes, which are unlikely to be the functional units in the brain (Glasser et al., 2016). Second, when applying these approaches, random parcellation would be visually similar to functional parcellation (Craddock et al., 2012). Third, when applying these approaches, random parcellation and functional parcellation tend to achieve nearly identical performances under different evaluation metrics, such as Dice coefficient and silhouette width (Craddock et al., 2012; Wang and Wang, 2016). The utility of such approaches is limited due to the above three problems. Therefore, to justify a parcellation approach, besides visually inspecting the generated clusters, it is necessary to compare the results obtained based on functional magnetic resonance imaging (fMRI) data to those obtained based on random data obtained using the same approach. If the two results are very close, then the parcellation approach encounters the above problems and is not reasonable, and vice versa.

To our knowledge, only few studies (Gordon et al., 2016; Parisot et al., 2016; Arslan et al., 2017; Gallardo et al., 2017) have demonstrated that their parcellations are better than corresponding random parcellations. Among these studies, Gordon et al. (2016) created null models by randomly rotating each hemisphere of the original parcellation, Parisot et al. (2016) and Arslan et al. (2017) created random parcellations by Poisson disk sampling, and Gallardo et al. (2017) created random parcellations by random region growing and random hierarchical clustering. All of these studies focused on parcellating the cortical surface rather than parcellating the brain volume. Our study focuses on volume-based analysis. Therefore, the strategies to generate random parcellations using the above approaches cannot be directly applied in our case. In this study, we generated random parcellations by applying parcellation approaches to random data. Except for the proposed GWC approach, two state-of-the-art whole-brain parcellation approaches, i.e., the Ncut approach (Craddock et al., 2012) and the SLIC approach (Wang et al., 2016) were compared in the experiments. By comparing these approaches to their corresponding random versions, we demonstrated that unlike the other two approaches, GWC does not rely heavily on spatial structures. We modified the Ncut and SLIC approaches to reduce their dependencies on spatial structures in order to avoid the above problems, and then treated the new approaches as competing approaches. Experimental results showed that GWC outperformed the two competing approaches under different evaluation metrics. In summary, our study verifies both the rationality and superiority of the proposed GWC approach.

## Materials and methods

### Participants and imaging data acquisition

In this study, we used publicly available data from the 1,000 Functional Connectomes Project (https://www.nitrc.org/projects/fcon_1,000/; Biswal et al., 2010). Specifically, we used the structural and resting-state fMRI data acquired from the first thirty-six subjects in the Beijing_Zang dataset of that project. The demographics and scanning parameters of this dataset can be found online.

### Preprocessing

The dataset was preprocessed using the Data Processing Assistant for Resting-State fMRI (DPARSF) (Yan and Zang, 2010), which was built on Statistical Parametric Mapping (SPM) (Friston et al., 1994). The preprocessing steps included discarding the first ten volumes; slice timing correction; motion correction; coregistration; segmenting the structural images; normalizing the functional images to the Montreal Neurological Institute (MNI) space at 4 × 4 × 4 mm^3^ resolution; smoothing with a 6-mm full width at half maximum (FWHM) Gaussian kernel; linear detrending; bandpass filtering with a passband of 0.01–0.08 Hz; regressing out nuisance covariates including six head motion parameters, autoregressive models of motion (the Friston 24-parameter model; Friston et al., 1996; Yan et al., 2013), and the mean time courses of white matter signal and cerebrospinal fluid signal. No subject was excluded due to excessive head motion under the excluding criteria of 2.0 mm and 2.0 degrees.

### Supervoxel generation

We applied SLIC (Achanta et al., 2012; Lucchi et al., 2012) to the preprocessed fMRI data to generate supervoxels for further clustering. SLIC has previously been used to perform whole-brain parcellation. In Wang et al. (2016), we applied SLIC to resting-state fMRI time series directly to perform individual subject-level parcellation. In Wang and Wang (2016), we applied SLIC to the features extracted by Ncut (Shi and Malik, 2000; Craddock et al., 2012) to perform group-level parcellation. The current study focuses on individual subject-level parcellation. We generated supervoxels using the SLIC approach in Wang et al. (2016), and then combined these supervoxels to perform parcellation.

### Feature extraction

After generating the supervoxels, we extracted multiple features from them. In 2D image segmentation tasks, typical visual features extracted from superpixels include color, texture, and shape (Cheng et al., 2011; Kong et al., 2015; Wang X. F. et al., 2015). Usually, color feature is characterized by mean color and color histogram, texture is characterized by local binary pattern (LBP) (Ojala et al., 2002), and shape is characterized by scale-invariant feature transform based bag-of-words (SIFT-BoW) (Lowe, 1999; Cheng et al., 2011). In this study, we extracted three kinds of features from the supervoxels, namely mean intensity, intensity histogram, and LBP. The number of bins of the intensity histogram was empirically set to 12. We also set this number to 6 and 18, and found that it hardly affected the parcellation performance. LBP in 3D space was calculated according to the proposition in Montagne et al. (2013), which only considers six nearest neighborhoods for encoding patterns. The number of bins for LBP was set to 10, as there were 10 groups of patterns in the LBP method we utilized.

### GWC

After extracting features from the supervoxels, we input these features into GWC to perform parcellation. Assume there are *N*_0_ voxels in the brain, the voxels are aggregated into *N* supervoxels by SLIC, and *M* features are extracted for each supervoxel. Let

$$\begin{array}{l} \begin{array}{c} \begin{array}{c} X=\left[x_{1},x_{2},\ldots ,x_{N}\right]\in R^{3\times N} \end{array} \end{array} \end{array}$$

denote the average coordinates of the supervoxels. Let

$$\begin{array}{l} \begin{array}{c} \begin{array}{c} Y^{\left(m\right)}=\left[y_{1}^{\left(m\right)},y_{2}^{\left(m\right)},\ldots ,y_{N}^{\left(m\right)}\right]\in R^{d_{m}\times N} \end{array} \end{array} \end{array}$$

denote the feature matrix of the *m*th feature of the supervoxels, *m* = 1, 2, …, *M*. The aim of GWC (Gao et al., 2016) is to find a graph *S* ∈ *R*^*N* × *N*^ that reflects the similarity between supervoxels based on spatial information and feature information. Meanwhile, *S* has exactly *K* connected components so that the supervoxels are combined into *K* clusters. This is achieved by formulating an optimization problem consisting of several parts that restrict the graph *S* to satisfy the desired properties. Similar ideas have previously been implemented to perform brain parcellation in Ryali et al. (2013) and Honnorat et al. (2015), with the aim of generating brain atlases that fit in different models.

An ideal graph *S* should reflect the spatial information as well as the feature information of the supervoxels, which can be formulated as

(1) $$\begin{array}{r} \begin{array}{c} \underset{s,a}{\min}g\left(X,S\right)+\lambda \underset{m}{\sum }\alpha_{m}h\left(Y^{\left(m\right)},S\right)+\beta f\left(S,\alpha \right), \end{array} \end{array}$$

where *g*(*X, S*) is the penalty function (also called cost function or energy function) that measures the smoothness between the graph *S* and the spatial information *X*, *h*(*Y*^(*m*)^, *S*) is the penalty function that measures the smoothness between the graph *S* and the *m*th feature *Y*^(*m*)^, *f*(*S*, α) is the regularization term of the target variables *S* and α, λ and β are tuning parameters, and

$$\begin{array}{l} \begin{array}{c} \begin{array}{c} \alpha ={\left[\alpha_{1},\alpha_{2},\ldots ,\alpha_{M}\right]}^{T}\in R^{M\times 1} \end{array}, \end{array} \end{array}$$

α_*m*_ is the *m*th element in the vector α and it determines the importance of the *m*th feature, *m* = 1, 2, …, *M*. The penalty function *g*(*X, S*) is defined as follows:

(2) $$\begin{array}{r} \begin{array}{c} g\left(X,S\right)=\underset{ij}{\sum }||x_{i}-x_{j}{||}_{2}^{2}s_{ij}, \end{array} \end{array}$$

where *x*_*i*_ and *x*_*j*_ denote the average coordinates of the voxels in the *i*th supervoxel and the *j*th supervoxel, respectively. This function ensures that supervoxels with small spatial distances have large weights on the corresponding edges in graph *S*, and vice versa. Similarly, the penalty function *h*(*Y*^(*m*)^, *S*) is defined as

(3) $$\begin{array}{r} \begin{array}{c} h\left(Y^{\left(m\right)},S\right)=\underset{ij}{\sum }||y_{i}^{\left(m\right)}-y_{j}^{\left(m\right)}{||}_{2}^{2}s_{ij}, \end{array} \end{array}$$

This function ensures that supervoxels with small feature distances have large weights on the corresponding edges in the graph *S*, and vice versa. Therefore, by combining the above two penalty functions, the graph *S* reflects both the spatial information and the feature information of the supervoxels. The regularization term *f*(*S*, α) is defined as follows:

(4) $$\begin{array}{r} \begin{array}{c} f\left(S,\alpha \right)=||S{||}_{F}^{2}+\gamma ||\alpha {||}_{2}^{2}, \end{array} \end{array}$$

where γ is a tuning parameter. Let

$$\begin{array}{l} \begin{array}{c} \begin{array}{c} S=\left[s_{1},s_{2},\ldots ,s_{N}\right]\in R^{N\times N} \end{array}, \end{array} \end{array}$$

where $s_{i}\in R^{N\times 1}$, *i* = 1, 2, …, *N*. The target variables *S* and α are further constrained as follows:

(5) $$\begin{array}{r} \begin{array}{c} s_{i}^{T}1=1,\;s_{i}\geq 0,\;i=1,2,\ldots ,N, \end{array} \end{array}$$

and

(6) $$\begin{array}{r} \begin{array}{c} \alpha^{T}1=1,\;\alpha \geq 0, \end{array} \end{array}$$

where **1** denotes a vector whose elements are ones and its length is not fixed. By combining the penalty functions (2) and (3), the regularization term (4), and the constraints (5) and (6), we can rewrite the optimization problem (1) as

(7) $$\begin{array}{l} \underset{S,\alpha }{\min}\sum_{ij}{\left\|x_{i}-x_{j}\right\|}_{2}^{2}s_{ij}+\lambda \sum_{mij}\alpha_{m}{\left\|y_{i}^{\left(m\right)}-y_{j}^{\left(m\right)}\right\|}_{2}^{2}s_{ij}\\ \;+\beta {\left\|S\right\|}_{F}^{2}+\beta \gamma {\left\|\alpha \right\|}_{2}^{2}\\ s.t.\;s_{i}^{T}1=1,\;s_{i}\geq 0,\;i=1,2,\ldots ,N\\ \;\alpha^{T}1=1,\;\alpha \geq 0. \end{array}$$

In order to combine the supervoxels into *K* clusters, the graphs *S* should contain exactly *K* connected components. According to Nie et al. (2014) and Gao et al. (2016), this can be achieved by introducing a new penalty function to problem (7), as follows:

(8) $$\begin{array}{l} \underset{S,\alpha ,Z}{\min}\;\underset{ij}{\sum }||x_{i}-x_{j}{||}_{2}^{2}s_{ij}+\lambda \underset{mij}{\sum }\alpha_{m}||y_{i}^{\left(m\right)}-y_{j}^{\left(m\right)}{||}_{2}^{2}s_{ij}\\ \;+\mu \underset{ij}{\sum }||z_{i}-z_{j}{||}_{2}^{2}s_{ij}+\beta ||S{||}_{F}^{2}+\beta \gamma ||\alpha {||}_{2}^{2}\\ s.t.\;s_{i}^{T}1=1,\;s_{i}\geq 0,\;i=1,2,\ldots ,N\\ \;\alpha^{T}1=1,\;\alpha \geq 0\\ \;Z^{T}Z=I,\;Z\in R^{N\times K}, \end{array}$$

where

$$\begin{array}{l} \begin{array}{c} \begin{array}{c} Z=\left[z_{1},z_{2},\ldots ,z_{K}\right]\in R^{N\times K} \end{array}, \end{array} \end{array}$$

μ is a sufficiently large constant and we set its value to 10^4^ in practice.

### The algorithm procedure

The optimization problem (8) was solved using the following iteration procedure. We first initialized α = 1/*M* and then initialized *S* based on the solution to problem (7). After that, we performed the following three steps in order. The first step was to update *Z* by fixing *S* and α, the second step was to update *S* by fixing α and *Z*, and the third step was to update α by fixing *Z* and *S*. The details of the updating steps are described below.

#### Updating Z

By fixing *S* and α, problem (8) becomes

(9) $$\begin{array}{l} \underset{Z}{\min}\sum_{ij}{\left\|z_{i}-z_{j}\right\|}_{2}^{2}s_{ij}\\ \;s.t.\;Z^{T}Z=I,\;Z\in R^{N\times K}. \end{array}$$

In order to solve this problem, we define a symmetric matrix

$$\begin{array}{l} S^{{\;}^{*}}=\frac{S^{T}+S}{2}. \end{array}$$

Let *D* be an *N* × *N* diagonal matrix whose *i*th diagonal element satisfies

$$\begin{array}{l} D\left(i,i\right)=\underset{j}{\sum }s_{ij}^{{\;}^{*}},\;i=1,2,\ldots N. \end{array}$$

Let *L* be a Laplacian matrix that satisfies

$$\begin{array}{l} L=D-S^{{\;}^{*}}. \end{array}$$

Then we have

$$\begin{array}{l} \underset{ij}{\sum }||z_{i}-z_{j}{||}_{2}^{2}s_{ij}=2tr\left(Z^{T}LZ\right). \end{array}$$

Problem (9) is equivalent to

(10) $$\begin{array}{l} \underset{Z}{\min}tr\left(Z^{T}LZ\right)\\ s.t.\;Z^{T}Z=I,\;Z\in R^{N\times K}. \end{array}$$

The optimal *Z* is formed by the *K* eigenvectors corresponding to the *K* smallest eigenvalues of *L*.

#### Updating S

By fixing α and *Z*, problem (8) becomes

(11) $$\begin{array}{l} \underset{S}{\min}\;\underset{ij}{\sum }||x_{i}-x_{j}{||}_{2}^{2}s_{ij}+\lambda \underset{mij}{\sum }\alpha_{m}||y_{i}^{\left(m\right)}-y_{j}^{\left(m\right)}{||}_{2}^{2}s_{ij}\\ \;+\mu \underset{ij}{\sum }||z_{i}-z_{j}{||}_{2}^{2}s_{ij}+\beta ||S{||}_{F}^{2}\\ s.t.\;s_{i}^{T}1=1,\;s_{i}\geq 0,\;i=1,2,\ldots ,N. \end{array}$$

To simplify problem (11), we define

(12) $$\begin{array}{r} p_{ij}=||x_{i}-x_{j}{||}_{2}^{2}+\lambda \underset{m}{\sum }\alpha_{m}||y_{i}^{\left(m\right)}-y_{j}^{\left(m\right)}{||}_{2}^{2}\\ +\mu ||z_{i}-z_{j}{||}_{2}^{2} \end{array}$$

*i, j* = 1, 2, …, *N*. *P* ∈ *R*^*N* × *N*^ is a constant matrix. Problem (11) can then be rewritten as

(13) $$\begin{array}{l} \underset{{\;}_{S}}{\min}\;\sum_{ij}p_{ij}s_{ij}+\beta {\left\|S\right\|}_{F}^{2}\\ s.t.\;s_{i}^{T}1=1,\;s_{i}\geq 0,\;i=1,2,\ldots ,N. \end{array}$$

The matrix *P* can be denoted by its columns as follows:

(14) $$\begin{array}{r} P=\left[p_{1},p_{2},\ldots ,p_{N}\right]\in R^{N\times N}, \end{array}$$

Since problem (13) is independent for different values of *i*, we separate it into a series of optimization problems as follows:

(15) $$\begin{array}{l} \underset{s_{i}}{\min}\;p_{i}^{T}s_{i}+\beta s_{i}^{T}s_{i}\\ s.t.\;s_{i}^{T}1=1,\;s_{i}\geq 0, \end{array}$$

*i* = 1, 2, …, *N*. Problem (15) is a quadratic programming problem. It has an optimal solution when β is a positive value. It can be rewritten as

(16) $$\begin{array}{l} \underset{s_{i}}{\min}\;{\left\|s_{i}+\frac{p_{i}}{2\beta }\right\|}_{2}^{2}\\ s.t.\;s_{i}^{T}1=1,\;s_{i}\geq 0. \end{array}$$

The solution to problem (16) is provided in the section titled Quadratic programming II in the Supplementary Materials. Suppose that the number of nonzero elements in *s*_*i*_ is *k*. It means that only *k* supervoxels can possibly be connected to the *i*th supervoxel. By fixing *k*, we can determine β and obtain the optimal *s*_*i*_. Tuning the parameter *k* is easier than tuning β since *k* is an integer and has an explicit meaning. Therefore, we chose to tune *k* rather than β in practice. In the initialization step, problem (7) with an initialized α was solved in the same manner as that used to solve problem (11).

#### Updating α

By fixing *Z* and *S*, problem (8) becomes

(17) $$\begin{array}{l} \underset{\alpha }{\min}\;\lambda \sum_{mij}\alpha_{m}{\left\|y_{i}^{\left(m\right)}-y_{j}^{\left(m\right)}\right\|}_{2}^{2}s_{ij}+\beta \gamma {\left\|\alpha \right\|}_{2}^{2}\\ s.t.\;\alpha^{T}1=1,\;\alpha \geq 0. \end{array}$$

Define

(18) $$\begin{array}{r} \;q={\left[q_{1},q_{2},\ldots ,q_{M}\right]}^{T}\in R^{M\times 1}, \end{array}$$

where

(19) $$\begin{array}{c} \begin{array}{c} \;q_{m}=\sum_{ij}{\left\|y_{i}^{\left(m\right)}-y_{j}^{\left(m\right)}\right\|}_{2}^{2}s_{ij}, \end{array} \end{array}$$

*m* = 1, 2, …, *M*. Then problem (17) can be simplified as

(20) $$\begin{array}{l} \underset{\alpha }{\min}\;\lambda q^{T}\alpha +\beta \gamma \alpha^{T}\alpha \\ s.t.\;\alpha^{T}1=1,\;\alpha \geq 0. \end{array}$$

It is a quadratic programming problem. And it has an optimal solution when βγ is a positive value. Problem (20) can be rewritten as

(21) $$\begin{array}{l} \underset{\alpha }{\min}\;{\left\|\alpha +\frac{\lambda q}{2\beta \gamma }\right\|}_{2}^{2}\\ s.t.\;\alpha^{T}1=1,\;\alpha \geq 0, \end{array}$$

where *q* is a constant vector, β is a constant value, and λ and γ are tuning parameters. When updating *S* by solving problem (16), we can obtain a β value for each *i*. We averaged the obtained β values and assigned the averaged result to the β in problem (21). Therefore, λ*q*/(2βγ) is a constant vector as a whole. The solution to problem (21) is provided in the section titled Quadratic programming I in the Supplementary Materials.

**Figure 1:**
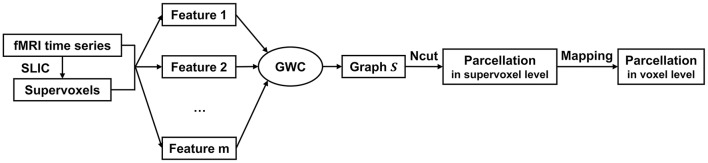
The algorithm procedure for the GWC approach. First, we applied SLIC directly to the fMRI time series to generate supervoxels. Next, we extracted multiple features from the supervoxels. By inputting these features into the GWC algorithm, we obtained the graph *S*. Then, we applied Ncut to *S* and obtained the parcellation at the supervoxel level. Finally, we mapped the parcellation to the voxel level. The parcellation at the voxel level was the final brain atlas.

The above updating steps were repeated until *S* was converged or the maximum number of iterations, i.e., 100, was reached. The graph *S* is theoretically guaranteed to contain exactly *K* connected components. To extract these components, we applied Ncut (Craddock et al., 2012; Wang et al., 2016) to graph *S*. This step generated cluster labels for the supervoxels. After that, we mapped the parcellation result from the supervoxel level to the voxel level. The parcellation result at the voxel level is the final brain atlas. Table 1 and Figure 1 summarize the algorithm procedure for the GWC approach.

**Table 1:** The algorithm procedure for the GWC approach.

**Input:** number of supervoxels, number of clusters, and parameters λ, γ, and *k*.
**Output:** parcellation result.
Generate supervoxels by SLIC.
Calculate the average coordinates and extract multiple features for the supervoxels.
**Apply GWC to calculate the graph S:**
Initialize α = 1/*M* and then initialize *S* based on the solution to problem (5).
**while** *S* is not converged and the maximum number of iterations is not reached **do**
Update *Z* by fixing *S* and α according to problem (7).
Update *S* by fixing α and *Z* according to problem (10).
Update α by fixing *Z* and *S* according to problem (14).
**end while**
Apply Ncut to *S* to generate cluster labels for the supervoxels.
Map the parcellation result from the supervoxel level to the voxel level.

### Graph normalization

Based on the definition of the matrix *P* in (12) and the definition of the vector *q* in (18–19), we observed that the average coordinates *X*, the features *Y*, and the eigenvectors *Z* are transformed into graphs. We defined the graph of the average coordinates *X* as follows:

$$\begin{array}{l} \begin{array}{c} {G_{ij}=||x_{i}-x_{j}||}_{2}, \end{array} \end{array}$$

*i, j* = 1, 2, …, *N*. The graphs of the features and the graphs of the eigenvectors were defined likewise. Then the matrix *P* was equal to the weighted sum of the squares of the graphs. To calculate the *m*th element of the vector *q*, i.e., *q*_*m*_, we first calculated the Hadamard product between the graph *S* and the square of the graph of the *m*th feature, and then summed all of the elements in the Hadamard product. Based on the optimization problems (16) and (21), we concluded that the average coordinates, features, and eigenvectors were in fact participating in GWC in the form of graphs.

These graphs were measured and computed on different scales. In order to adjust them to a notionally common scale, a normalization procedure was performed on each graph. Specifically, we divided all of the elements in each graph by the maximum value in that graph. We also attempted to normalize the average coordinates, features, and eigenvectors instead of normalizing their graphs. However, this strategy reduced the clustering performance of GWC in practice. Therefore, we performed normalization on graphs in our experiments.

### Tuning parameters

#### The number of supervoxels

The number of supervoxels was empirically set to 1,000. If this number is too large, there would be only few voxels in each supervoxel. In this case, the impact of the supervoxel method would be limited. If this number is too small, there would be only few supervoxels combined in each cluster in the final brain atlas, especially when the cluster number is large. This would deteriorate the performance of the GWC approach in practice.

#### The number of clusters

To the best of our knowledge, there is no optimal cluster number. The initialized cluster number was set to [25:25:500] in order to generate parcellations with multiple granularities. This range covers the cluster numbers of the brain atlases in most of the latest studies (Fan et al., 2016; Glasser et al., 2016; Gordon et al., 2016; Arslan et al., 2017; Schaefer et al., 2017).

#### The parameter λ

The parameter λ was set to 0.1 empirically. This parameter determines the weight of visual features in the optimization problem of GWC, and therefore indirectly determines the weight of spatial information in that problem. By setting it to be a small value, the weight of spatial information would be large. This can guarantee the spatial contiguity of the parcellation result. However, λ should not be too small, or the parcellation approach would rely heavily on spatial structures.

#### The parameter Γ

The parameter γ was set to 1 empirically. This parameter determines the weight of the regularization term α (Gao et al., 2016). When γ is set to a small value, GWC depends on the performance of the optimal feature. When γ is set to a large value, different features have similar weights. The performance of GWC is not sensitive to γ in image segmentation tasks (Gao et al., 2016). We found that this rule also applied to the whole-brain parcellation tasks in our study. Therefore, we set the value of γ to 1.

#### The parameter k

**Figure 2:**
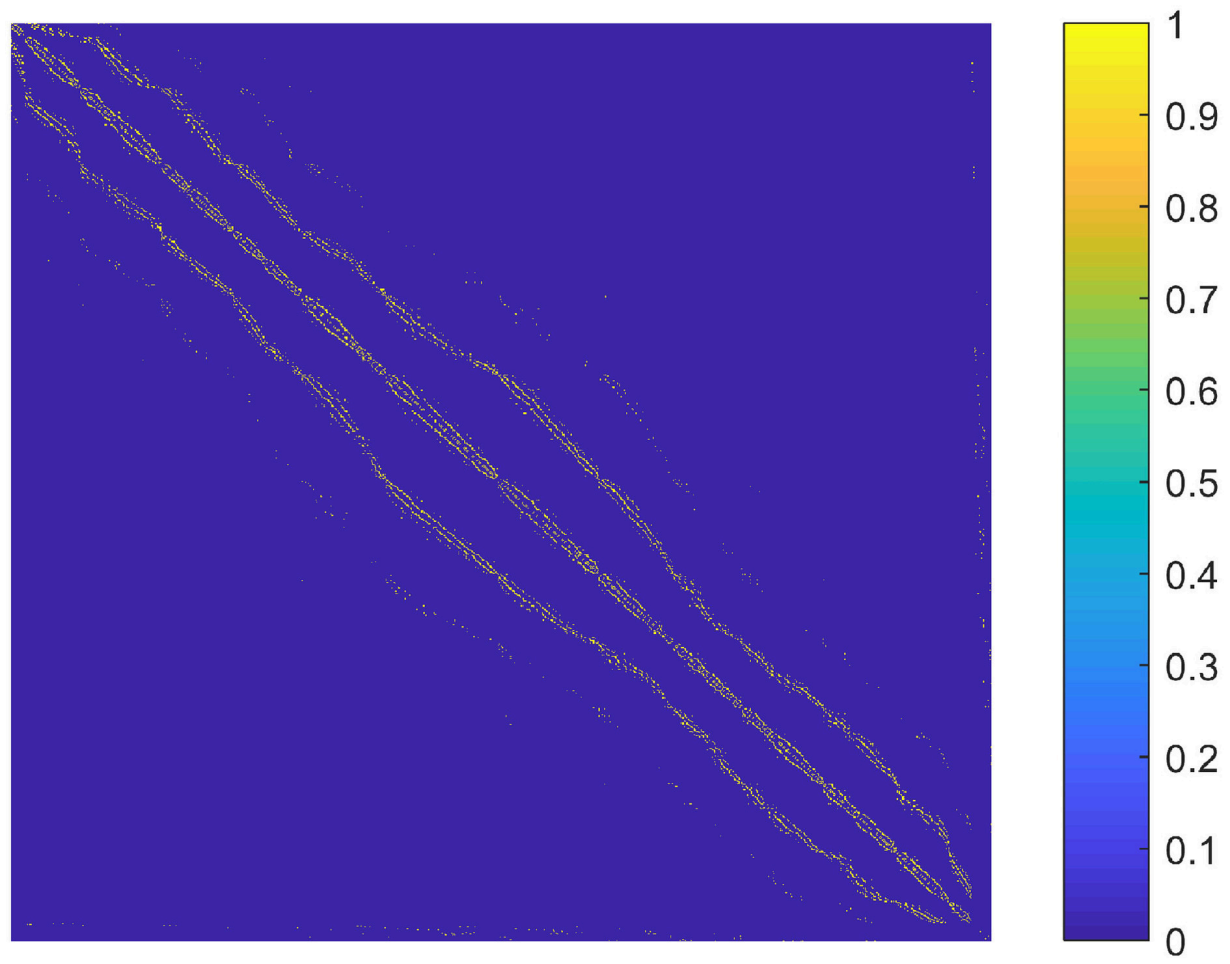
A graph illustrating the spatial relationship between supervoxels of the first subject. Each node (row and column) represents a supervoxel. If two supervoxels are spatially connected, then the corresponding element in the graph is set to one. This value is set to zero otherwise.

The parameter *k* is the number of nonzero elements in each column of the graph *S*. We determined *k* by adapting the idea of the second sparsifying scheme in Wang and Wang (2016). To choose an appropriate *k* value, we constructed a graph *S*_0_ that reflected the spatial relationship between the supervoxels, and assumed that the sparse rate of *S* was close to that of *S*_0_. To be specific, *S*_0_ was an *N* × *N* adjacency matrix formulated by setting the element *s*_*ij*_ to one if supervoxels *i* and *j* were spatially connected, and to zero otherwise. Here, two supervoxels were spatially connected meant that we could find at least one voxel from each of them so that the two voxels were in the 26-connected neighborhoods of each other in 3D space. Then *k* was estimated as the average number of nonzero elements in the columns of *S*_0_. The graph *S*_0_ for the first subject is shown in Figure 2. It reflects the spatial relationship between supervoxels. Correspondingly, the spatial constraint in Wang and Wang (2016) reflects the spatial relationship between voxels. Therefore, the two are very similar. The resultant value of *k* was nine for all subjects. Therefore, we set *k* to nine in our experiments.

### Evaluation criteria

To evaluate the parcellation results, we used three criteria, i.e., spatial contiguity, functional homogeneity, and reproducibility (Wang and Wang, 2016; Wang et al., 2016). It is worthwhile to mention that there is no gold standard for the evaluation of a brain parcellation (Thirion et al., 2014; Eickhoff et al., 2015) and that the above evaluation criteria have some inherent limitations (Wang and Wang, 2016). To the best of our knowledge, the most credible way to evaluate a brain parcellation is manually comparing it to multi-modal areal features by experienced neuroanatomists (Blumensath et al., 2013; Glasser et al., 2016). This is beyond the scope of our study. Nevertheless, the aforementioned three criteria are widely used. Therefore, we used these criteria to evaluate the parcellation results in our experiments.

For spatial contiguity, we identified the spatially discrete regions that belonged to the same cluster in a brain atlas, and assigned a unique label to each region. The increased cluster number was termed the spatial discontiguity index. A small result indicates that the clusters in the brain atlas has good spatial contiguity.

For functional homogeneity, we first averaged similarities across all pairs of voxels within each cluster in a brain atlas, and then averaged the obtained results across clusters. The final result is the functional homogeneity of the brain atlas. Assume that there are *N*_0_ voxels being parcellated into *K* clusters in a brain atlas; the voxel number in the *k*th cluster *C*_*k*_ is *n*_*k*_, *k* = 1, 2, …, *K*; and the similarity between voxels *i* and *j* is *s*_*ij*_ which equals the Pearson correlation coefficient in our study, *i, j* = 1, 2, …, *N*_0_. The average similarity within the *k*th cluster is

$$\begin{array}{c} a\left(k\right)=\frac{1}{n_{k}\left(n_{k}-1\right)}\sum_{i,j\in C_{k},\;i\neq j}s_{ij}. \end{array}$$

Then the functional homogeneity of the brain atlas is calculated by averaging the similarities across clusters, as follows:

$$\begin{array}{l} \begin{array}{c} \frac{1}{K}\overset{K}{\underset{k=1}{\sum }}a\left(k\right). \end{array} \end{array}$$

Clusters that contain only one voxel are omitted in the calculation. To avoid circular analysis, we trained an atlas on one subject and calculated the functional homogeneity based on this atlas and the resting-state fMRI data of the remaining subjects. A large result indicates good functional homogeneity.

To determine reproducibility, we calculated Dice coefficient (Dice, 1945) between two brain atlases that were independently generated from two subjects when the initialized cluster number was fixed. The average cluster sizes of the two brain atlases were very close. Prior to calculating the Dice coefficient, we calculated an adjacency matrix for each brain atlas. An adjacency matrix *A* is an *N*_0_ × *N*_0_ symmetric matrix that is calculated by setting its elements *a*_*ij*_ to one if voxels *i* and *j* belong to the same cluster in the brain atlas, and to zero otherwise. The Dice coefficient between two adjacency matrices *A* and *B* derived from two atlases is

$$\begin{array}{l} \frac{2|A\cap B|}{\left|A|+|B\right|}, \end{array}$$

where |·| denotes the number of ones in an adjacency matrix, and *A*∩*B* denotes the intersection of the two adjacency matrices. The intersection of two matrices, i.e., *C* = *A*∩*B*, is defined as follows: *C*_*ij*_ is set to one if and only if *A*_*ij*_ and *B*_*ij*_ are both equal to one, and *C*_*ij*_ is set to zero otherwise. A large Dice coefficient indicates good reproducibility.

### The competing approaches

In this study, we focused on individual subject-level parcellation. The competing approaches included the Ncut approach (Shi and Malik, 2000; Yu and Shi, 2003) and the SLIC approach (Achanta et al., 2012). The Ncut approach has been used to find resting-state networks (van den Heuvel et al., 2008), to subdivide regions of interest (Shen et al., 2010), and to parcellate the whole brain (Craddock et al., 2012; Shen et al., 2013). The SLIC approach has been used to parcellate the whole brain (Wang and Wang, 2016). Most of these studies focused on group-level parcellation. In Wang et al. (2016), the Ncut and SLIC approaches were applied as individual subject-level parcellation approaches, and were carefully compared.

The Ncut-based parcellation approaches (Craddock et al., 2012; Shen et al., 2013) rely heavily on spatial structures (Blumensath et al., 2013; Gordon et al., 2016; Wang and Wang, 2016). This is due to the spatial constraint in weight definition. The SLIC approach is also subject to this problem since it places great emphasis on spatial structures by initializing an ideal geometric pattern, integrating the spatial distance into the unified distance, and searching in a local space (Wang and Wang, 2016; Wang et al., 2016). Therefore, the utility of these approaches is limited. In this study, the proposed GWC approach incorporates spatial structures by SLIC in the supervoxel generation procedure, and by the penalty function of spatial distances in Equation (2). Therefore, the GWC approach might also encounter the aforementioned problem.

To determine whether a parcellation approach relies heavily on spatial structures, we applied the approach to fMRI data and random data, and then compared the obtained results. We will show that the Ncut and SLIC approaches rely heavily on spatial structures, and that the proposed GWC approach does not. To improve the two competing approaches, we attempted to weaken their dependence on spatial structures. This enabled us to make fair comparisons among the Ncut, SLIC, and GWC approaches.

For the Ncut approach, a typical weight is defined as follows. Suppose there are *N*_0_ voxels in the brain, *v*_*i*_ is the fMRI time course of the *i*th voxel, and *u*_*i*_ is the coordinates of the *i*th voxel in the MNI space, *i* = 1, 2, …, *N*_0_. The weight on the edge connecting voxels *i* and *j* is defined as

(22) $$w_{ij}=\{\begin{array}{cc} corr\left(v_{i},\;v_{j}\right)\;if & {\left\|u_{i}-u_{j}\right\|}_{2}\;\leq \sqrt{3}\\ 0 & otherwise, \end{array}$$

where *corr*(*v*_*i*_, *v*_*j*_) denotes the Pearson correlation coefficient between *v*_*i*_ and *v*_*j*_. This definition differs from that in Craddock et al. (2012) by taking out the hard threshold 0.5. Negative weights are often handled in graph analysis (Hallquist and Hillary, 2018), because some graph metrics still need to be defined or adapted when negative edges are present (Rubinov and Sporns, 2011). However, this is not the case in brain parcellation since the parcellation approaches in our study are feasible when negative and weak weights are present. Wang and Wang (2016) have demonstrated that taking out the hard threshold hardly affects the parcellation performances. Therefore, we did not include the hard threshold in (22). We will show that the Ncut approach with the weight in (22) relies heavily on the spatial structure introduced by spatial constraint.

To modify the Ncut approach (Craddock et al., 2012), we changed the weighting function to reduce its dependency on spatial structure. Gaussian functions are common choices for defining weight matrix (Shen et al., 2013; Cheng et al., 2014). Here we adapted the weight definition proposed in Shi and Malik (2000) by reserving the Gaussian function of functional distance and spatial distance, and removing the spatial constraint. That is,

(23) $$\begin{array}{r} \begin{array}{c} w_{ij}=e^{-\frac{||v_{i}-v_{j}{||}_{2}^{2}}{\sigma_{v}^{2}}-\frac{||u_{i}-u_{j}{||}_{2}^{2}}{\sigma_{u}^{2}}}, \end{array} \end{array}$$

where σ_*v*_ and σ_*u*_ are tuning parameters set to the median of all functional distances and spatial distances, respectively. Spatial information is incorporated in this definition, but is much weaker than the spatial constraint in (22). Therefore, the generated brain atlases are more meaningful. Since we did not apply a spatial constraint or a threshold to this weight matrix, it is a dense matrix and would consume significant computational resources. Note that other weighting functions might also be used. We used the weighting function defined in (23) because it was sufficient for our purpose.

For the SLIC approach (Wang et al., 2016), the unified distance between voxels *i* and *j* is defined as

(24) $$\begin{array}{r} \begin{array}{c} d_{ij}=\sqrt{\frac{||v_{i}-v_{j}{||}_{2}^{2}}{m^{2}}+\frac{||u_{i}-u_{j}{||}_{2}^{2}}{S^{2}}}, \end{array} \end{array}$$

where *m* and *S* are tuning parameters that normalize the functional distance and the spatial distance, respectively. The parameter *S* is set to the average length of the supervoxels. For the parameter *m*, we will show that a value of 40, which was used in Wang et al. (2016), is too large. This renders the parcellation results heavily reliant on spatial structures.

To modify the unified distance, we used a smaller *m* value, which increased the weight of functional distance in the unified distance and weakened the influence of spatial structures on the parcellation results. The *m* value was set to 10 because when *m* equals 10, the normalized functional distance ||*v*_*i*_ − *v*_*j*_||_2_/*m* and the normalized spatial distance ||*u*_*i*_ − *u*_*j*_||_2_/*S* have similar weights in the unified distance. To generate supervoxels for GWC using SLIC, the parameter *m* in SLIC was also set to 10. This allowed us to make a fair comparison between the modified SLIC approach and the GWC approach when they were applied as whole-brain parcellation approaches.

Other than the above modifications, the Ncut and SLIC approaches applied in this study were the same as those used in Wang et al. (2016). More details regarding the two approaches can be found in Craddock et al. (2012) and Wang and Wang (2016).

Although the weight in (23) and the unified distance in (24) are closely related, the strategies to select parameters for these values are quite different. This is because the Ncut and SLIC approaches differ substantially. One of the major differences is that Ncut is globally operated on a brain graph while SLIC is locally operated on supervoxels. Therefore, the parameters of the two approaches are tuned differently.

### Random parcellation

To determine whether a parcellation approach relies heavily on spatial structures, we compared its resulting parcellations to random parcellations using different evaluation metrics. In this study, random parcellations were generated by applying parcellation approaches to random data. For the Ncut approach, we randomly permutated the weight matrices calculated by Equations (22) and (23) to form random weight matrices, and then applied Ncut to the random weight matrices to perform parcellation. For the SLIC and GWC approaches, we randomly permutated the fMRI time series within the brain and then applied SLIC or GWC to the random fMRI time series to perform parcellation. In other words, to generate random parcellations, the Ncut approach was applied to random weight matrices while the SLIC and GWC approaches were applied to random fMRI time series. The random parcellation approaches corresponding to Ncut, SLIC, and GWC are referred to as random Ncut, random SLIC, and random GWC below.

The random parcellations were likewise evaluated by the three evaluation metrics. Note that functional homogeneity relies not only on the generated brain atlas, but also on the fMRI data from the subjects that did not participate in generating the atlas. Since there is no similarity between two random datasets, using random fMRI data to calculate homogeneity would result in a value close to zero. Therefore, we used original fMRI data rather than random fMRI data to calculate functional homogeneity for random parcellations. The other two evaluation metrics, i.e., spatial discontiguity index and Dice coefficient, were calculated only based on random parcellations.

## Results

### GWC

We applied the GWC approach to resting-state fMRI data from 36 subjects to perform individual subject-level parcellation. For each parcellation approach, each subject, and each cluster number, one brain atlas was generated using GWC. Spatial discontiguity index was calculated for each brain atlas, and then averaged across subjects. Functional homogeneity was calculated based on the brain atlas of one subject and the resting-state fMRI data of the remaining subjects. It was then averaged across subjects. To calculate reproducibility, we randomly chose one hundred pairs of subjects, calculated the Dice coefficient between the brain atlases of each pair of subjects, and then averaged the results across the one hundred pairs to obtain the mean Dice coefficient. We did not use all pairs of subjects to calculate the Dice coefficient because it was computationally expensive. Except for the group-averaged results, standard deviations were calculated to quantify the inter-individual variability for spatial contiguity and functional homogeneity, and to quantify the inter-pair variability for reproducibility.

**Figure 3:**
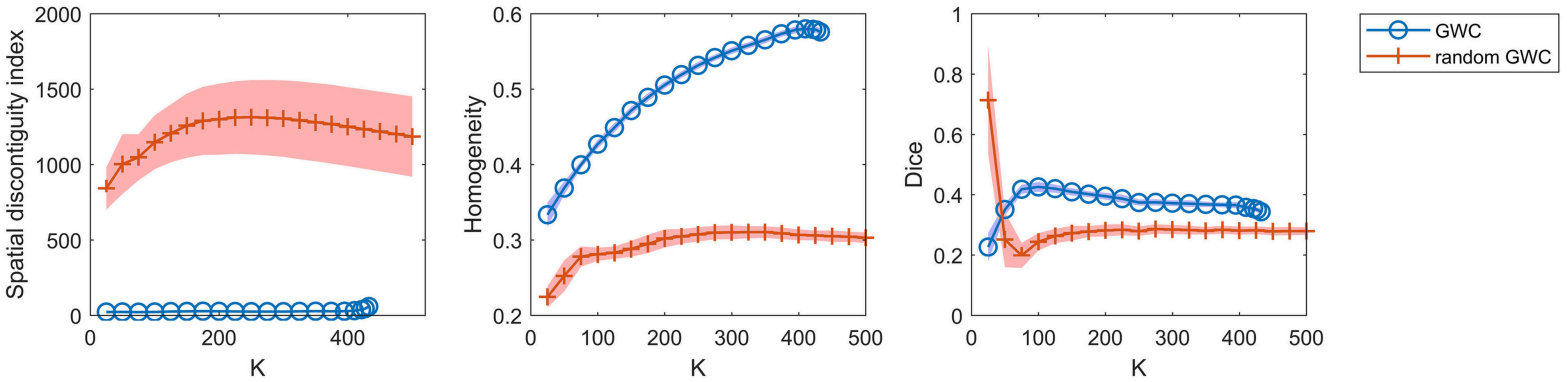
The results of the different evaluation metrics for the GWC approach and the random GWC approach. The three columns correspond to spatial discontiguity index, functional homogeneity, and Dice coefficient in order. The markers and shaded areas represent the mean plus/minus a standard deviation of the results. These evaluation metrics are plotted against the averaged actual cluster number which is denoted by K. A shared legend for the three subfigures is placed on the right side. Note that the shaded areas for some curves are very small.

To demonstrate that the GWC approach does not rely heavily on spatial structures, we compared its parcellations to the corresponding random parcellations using the three evaluation metrics. In other words, we compared between the GWC approach and the random GWC approach. Figure 3 shows the results of this comparison. The three evaluation metrics were plotted against the averaged actual cluster number. The initialized cluster numbers of the blue circles were [25:25:500], as presented from left to right in order in each subfigure. When the initialized cluster number was smaller than 400, the actual cluster number and the initialized cluster number were nearly equal. When the initialized cluster number was larger than 400, the actual cluster number was much smaller than the initialized cluster number. In summary, GWC behaves abnormally when the initialized cluster number is larger than 400. This is because only a few supervoxels are combined in each cluster when the initialized cluster number is larger than 400 given that the supervoxel number is 1,000, which negatively affects the performance of GWC. This problem does not occur when using random GWC, mainly because the supervoxels and the clusters generated by random GWC are rather spatially discontiguous. The results of GWC with initialized cluster number larger than 400 were not favorable when assessed using the three evaluation metrics. Therefore, we mainly considered the results with initialized cluster number smaller than 400 in the following experiments.

With the exception of one outlier, i.e., the Dice coefficient when the cluster number was 25, the results of GWC were much better than the results of random GWC. This indicates that the proposed GWC approach does not rely heavily on spatial structures.

We had repeated the random GWC approach multiple times and found that the results were rather robust with different randomizations. The random versions of Ncut, SLIC, and their aforementioned variants behaved similarly in our experiments. Therefore, the randomization steps in all of these random parcellation approaches could be set arbitrarily. We thus only displayed one result from the multiple computations.

### Tuning parameters

We then investigated the influences of the four parameters on the GWC approach. It is difficult to tune four parameters simultaneously. Therefore, we changed one parameter at a time and then checked how the parcellation performances were affected by each parameter.

**Figure 4:**
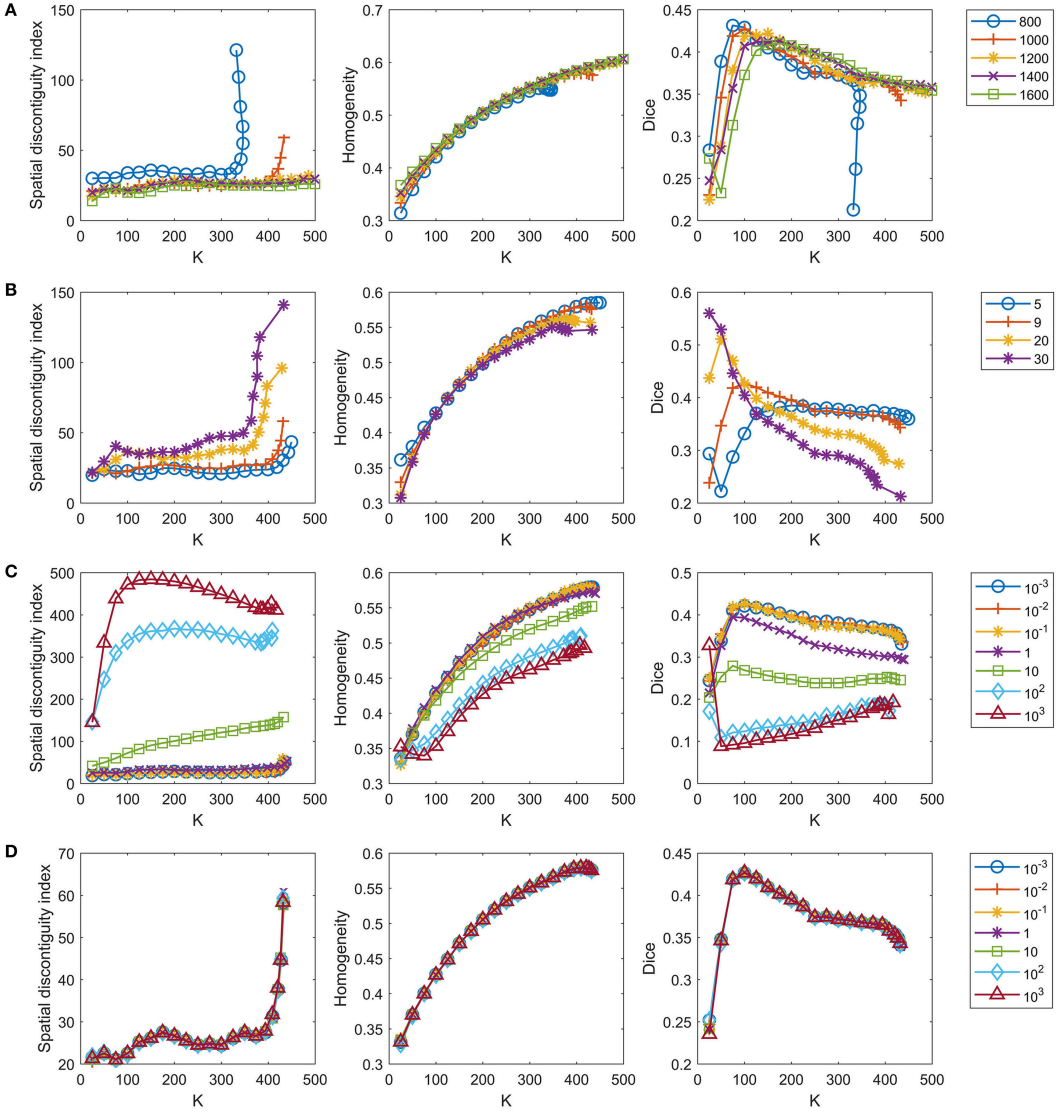
Tuning parameters for the GWC approach. One parameter is tuned in each row while the remaining parameters are fixed at the default settings. The four rows represent tuning of **(A)** the number of supervoxels, **(B)** parameter *k*, **(C)** parameter λ, and **(D)** parameter γ in order. We do not display the standard deviations for the curves because the mean results were sufficient for parameter selection.

The number of supervoxels was set to 1000 by default. We tested several values near 1000 to check whether setting this number to 1000 was appropriate. Figure 4A shows the results when the number of supervoxels was set to [800:200:1600]. The spatial contiguity improved when the supervoxel number increased. The functional homogeneity tended to increase with increasing supervoxel number, but the magnitudes of the changes were trivial. The Dice coefficient tended to decrease with increasing supervoxel number when the cluster number was smaller than 150, but tended to increase with increasing supervoxel number when the cluster number was larger than 150. To achieve balance, we fixed the supervoxel number to 1,000 in our experiments.

The parameter *k*, i.e., the number of nonzero elements in the columns of *S*, was set to 9 by default, as discussed in section The Parameter **k**. We tested three values near 9, i.e., 5, 20, and 30, to check whether setting *k* to 9 was appropriate. Figure 4B shows the results when *k* = [5, 9, 20, 30]. The spatial contiguity and functional homogeneity generally became worse when *k* increased. For the reproducibility, when the cluster number was larger than 25 and smaller than 225, the Dice coefficients when *k* = 5 were much smaller than those when *k* = 9. Generally speaking, *k* = 9 was the appropriate choice.

The parameter λ was set to 0.1 by default. As there was no prior knowledge regarding this parameter, we tuned it in a large range to determine whether the value 0.1 was appropriate, as in Gao et al. (2016). Figure 4C shows the results when λ = [10^−3^, 10^−2^, 10^−1^, 1, 10, 10^2^, 10^3^]. The results of the three evaluation metrics generally improved when λ decreased. When λ was smaller than 0.1, the differences between the results corresponding to different λ values were subtle. On the other hand, as discussed before, λ should not be too small, or the parcellation approach would rely heavily on spatial structures. Therefore, the optimal λ should be as large as possible while achieving good parcellation performances. With the above two considerations in mind, λ = 0.1 was determined to be a suitable choice.

The parameter γ was set to 1 by default. We tuned this parameter in a large range, as we tuned λ. Figure 4D shows the results when γ = [10^−3^, 10^−2^, 10^−1^, 1, 10, 10^2^, 10^3^]. The curves corresponding to different γ values overlapped with each other. This indicates that the γ value hardly affected the parcellation performances, which is consistent with the findings of Gao et al. (2016). Therefore, we set γ to 1 by default.

### Competing approaches

**Figure 5:**
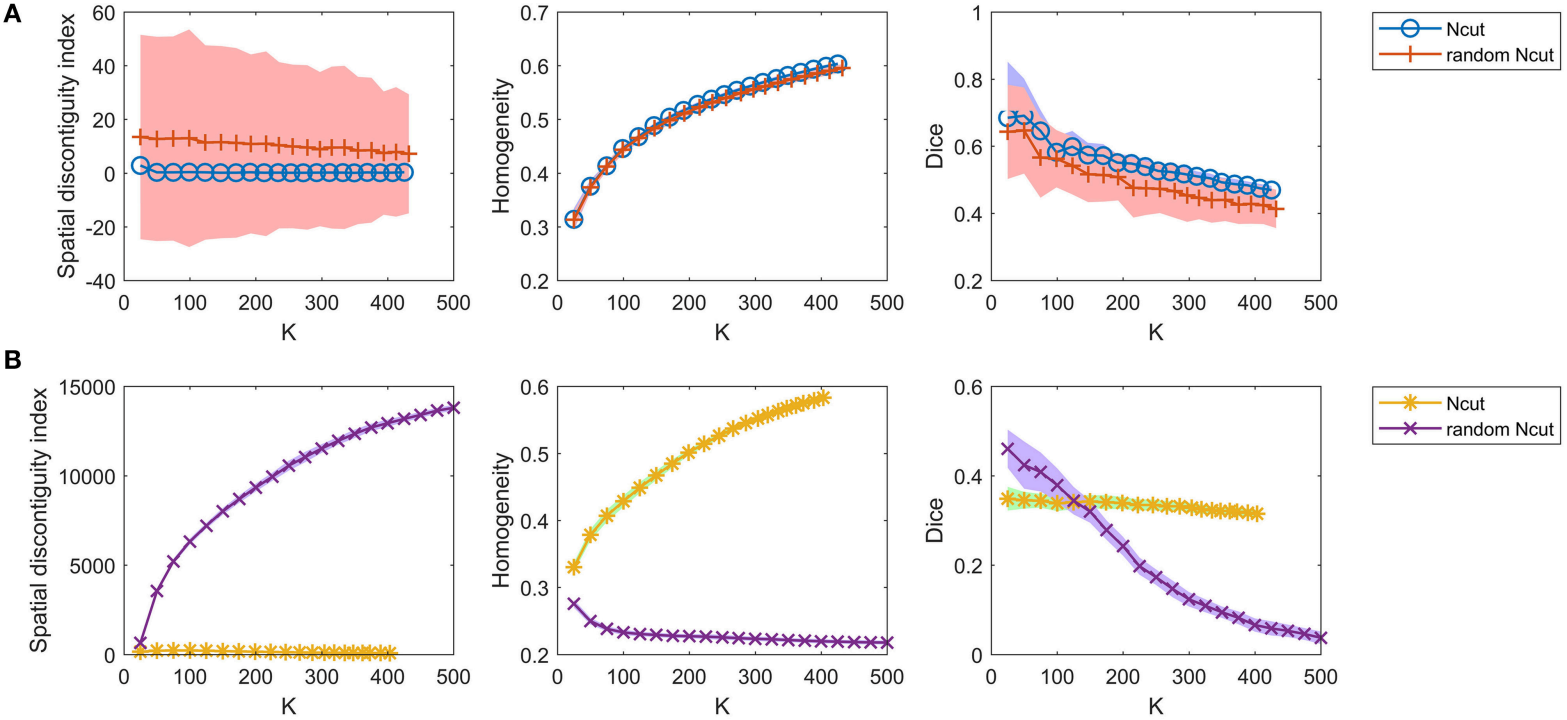
The results of the different evaluation metrics for the Ncut approach and the random Ncut approach. The two rows represent the results of **(A)** the two approaches using the weight in Equation (22), and **(B)** the two approaches using the weight in Equation (23).

For the competing approaches, we first investigated the Ncut and random Ncut approaches using the weight in Equation (22). The results are shown in Figure 5A. The performances of the random Ncut approach were only slightly worse than those of Ncut. This indicates that the Ncut approach with the weight in (22) relies heavily on spatial structures.

Next, we investigated the Ncut and random Ncut approaches with the weight in (23). The results are shown in Figure 5B. The performances of random Ncut were much worse than those of Ncut, with the exception of the Dice coefficients when the cluster number was no larger than 100. This demonstrates that the Ncut approach with the weight in (23) does not rely heavily on spatial structures. It is thus a more reasonable parcellation approach.

**Figure 6:**
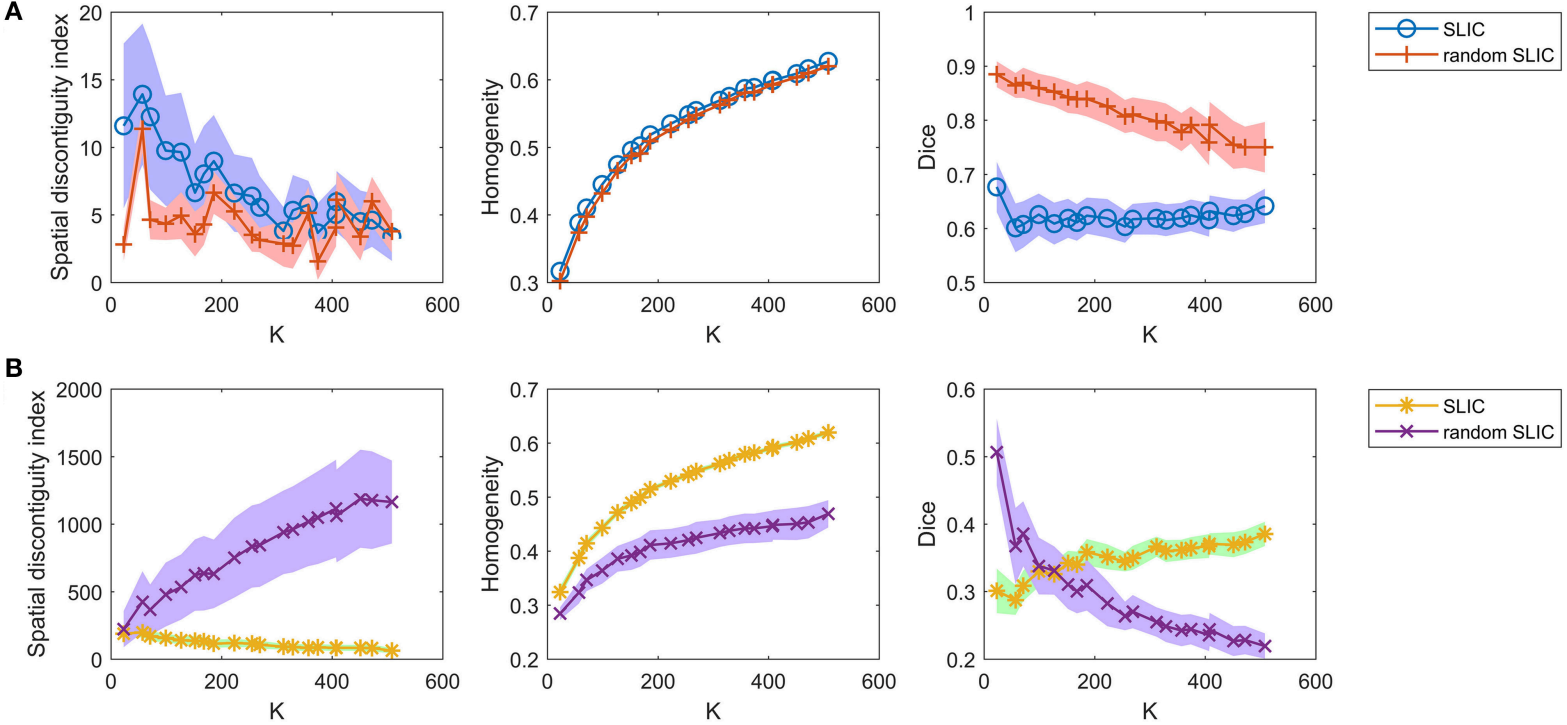
The results of the different evaluation metrics for the SLIC approach and the random SLIC approach. The two rows represent the results of **(A)** the two approaches with *m* = 40, and **(B)** the two approaches with *m* = 10.

Then, we investigated the SLIC and random SLIC approaches with *m* = 40. The results are shown in Figure 6A. For functional homogeneity, the two approaches were close. For spatial contiguity and reproducibility, random SLIC even performed better than SLIC. These results indicate that SLIC with *m* = 40 relies heavily on spatial structures.

Finally, we investigated the SLIC and random SLIC approaches with *m* = 10. The results are shown in Figure 6B. For spatial contiguity and functional homogeneity, SLIC performed much better than random SLIC. For reproducibility, SLIC performed better than random SLIC when the cluster number was larger than 150. These results demonstrate that SLIC with *m* = 10 does not rely heavily on spatial structures. It is therefore more reasonable than SLIC with *m* = 40.

Since the parameter *m* greatly influences the SLIC and random SLIC approaches, it is meaningful to test the two approaches when *m* is set to other values. The results are shown in Supplementary Figures 1–6. When *m* was smaller than 10, the performances of the two approaches were rather bad. When *m* was larger than 10, the differences in performances between SLIC and random SLIC diminished with increasing m values. These results indicate that SLIC relies heavily on spatial structures when the *m* value is larger than 10. Therefore, using *m* = 10 is an appropriate choice for the SLIC approach.

Note that the Ncut approach with the weight in (22) outperformed the Ncut approach with the weight in (23), and that the SLIC approach with *m* = 40 outperformed the SLIC approach with *m* = 10, especially in terms of spatial contiguity and reproducibility. This is mainly due to the fact that the Ncut approach with the weight in (22) and the SLIC approach with *m* = 40 rely heavily on spatial structures. Modifying the two approaches to avoid the problem in fact leads to sacrifices in parcellation performances. Nevertheless, the sacrifices are worthwhile.

### Comparison

Available online at: We have demonstrated that the three approaches, i.e., GWC with default parameters, Ncut with the weight in (23), and SLIC with *m* = 10, do not rely heavily on spatial structures. Therefore, they are reliable parcellation approaches. The three approaches are referred to as GWC, Ncut, and SLIC for brevity in the following experiments.

**Figure 7:**
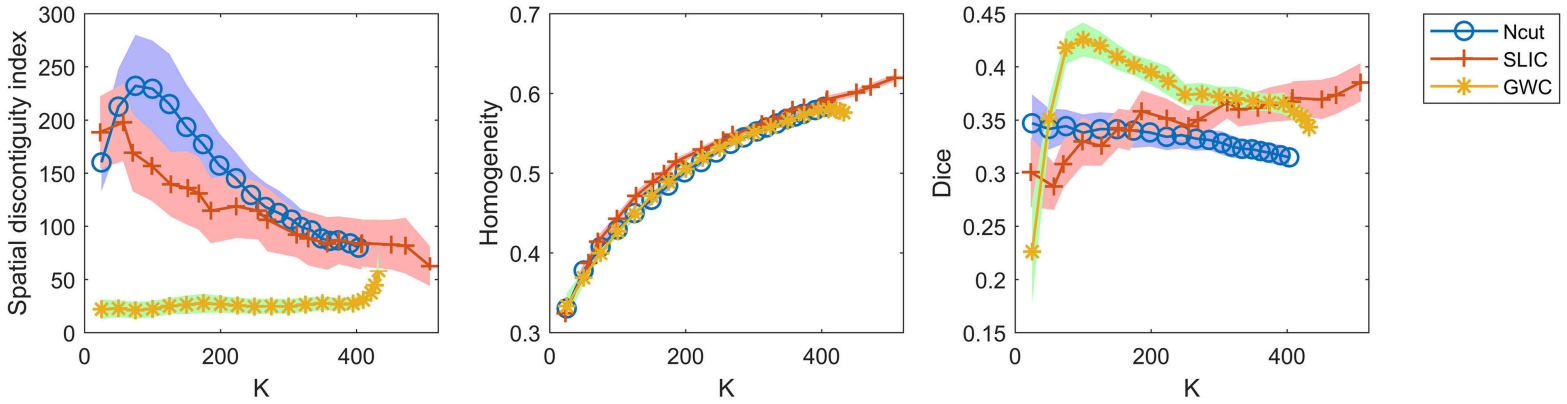
The results of the different evaluation metrics for the Ncut, SLIC, and GWC approaches.

A comparison of the three approaches is shown in Figure 7. As some curves were overlapping, we plotted the results in separate figures to reveal the concealed details, as shown in Supplementary Figure 7. For spatial contiguity, GWC greatly outperformed Ncut and SLIC. For functional homogeneity, the three approaches achieved similar performances. For reproducibility, GWC outperformed Ncut and SLIC when the initialized cluster number was larger than 50 and smaller than 400, and the best average result was obtained by GWC with an initialized cluster number of 100. Generally, GWC performed better than Ncut and SLIC. It is worthwhile to mention that SLIC outperformed Ncut with the exception of the Dice coefficients when the cluster number is smaller than 150.

**Figure 8:**
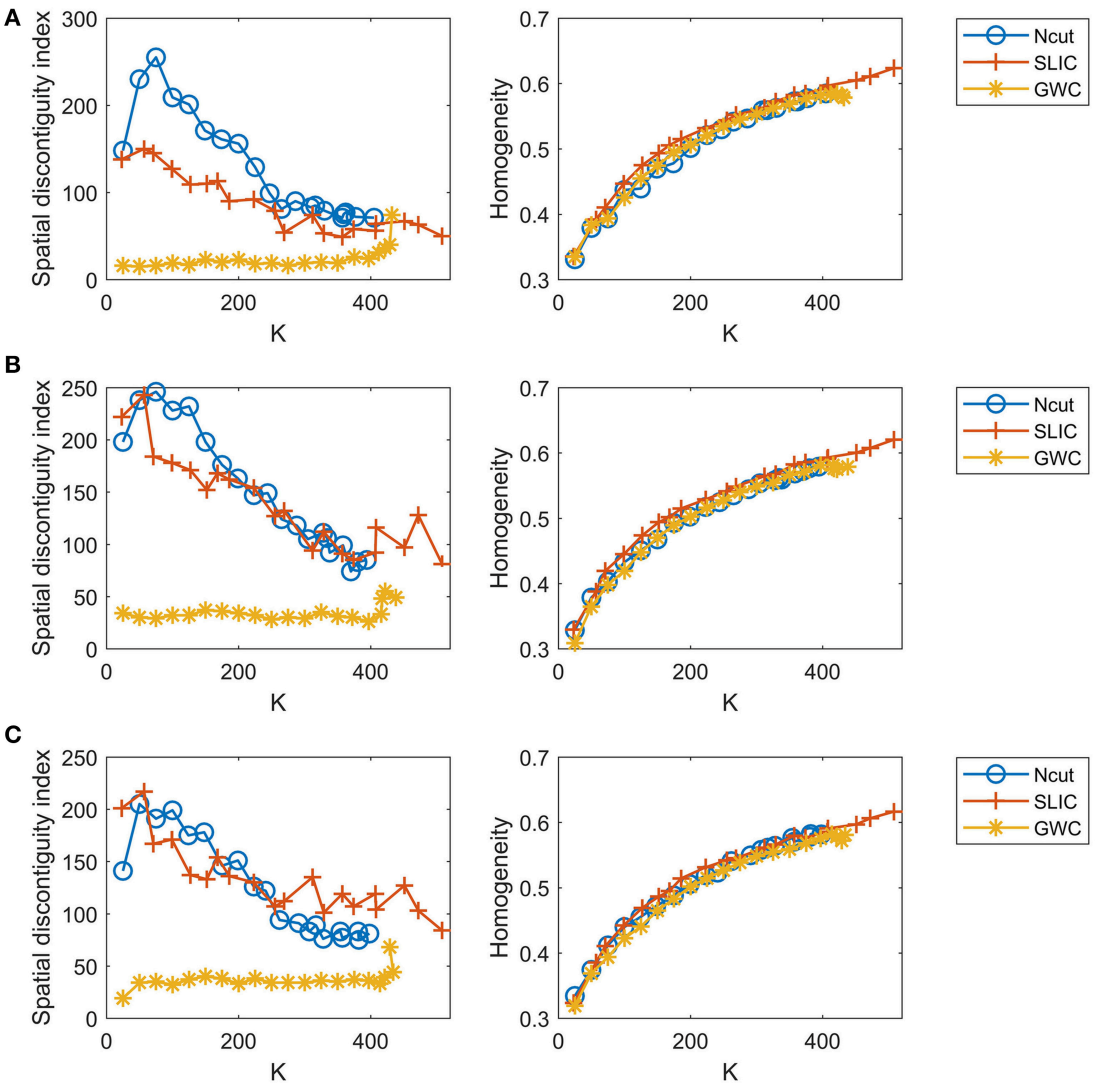
The results of spatial contiguity and functional homogeneity for the Ncut, SLIC, and GWC approaches. Each row represents the results of one subject. **(A)** Subject 1. **(B)** Subject 2. **(C)** Subject 3.

Since the three approaches are subject-level approaches, it is also meaningful to make comparisons among these approaches at the individual subject level. Figure 8 shows the results of spatial contiguity and functional homogeneity for the first three subjects. These results were generally consistent with the group-averaged results, which further demonstrated the superiority of the GWC approach. Moreover, the results of the three subjects were different from each other, which demonstrated the inter-subject variability. Therefore, GWC is an appropriate individual subject-level parcellation approach.

**Figure 9:**
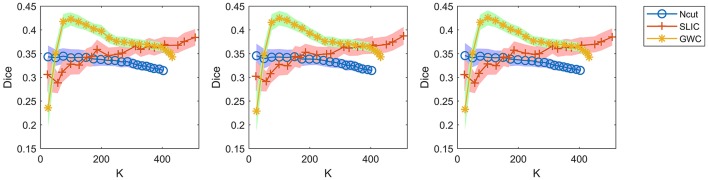
The results of reproducibility for the Ncut, SLIC, and GWC approaches. The first two columns show the results calculated based on two other random selections of subject pairs. The third column shows the results calculated based on all subject pairs.

When evaluating reproducibility, we randomly selected only one hundred pairs of subjects and averaged the Dice coefficients across these pairs. Since there were 36 subjects in our experiments, there were 36 × (36−1)/2, i.e., 630, pairs of subjects in total. We did not calculate the Dice coefficients based on all of these pairs because it was time-consuming. It was thus very important to guarantee that random sampling did not greatly alter the results of the Dice coefficients. To address this issue, we re-calculated the Dice coefficients in Figure 7 by using two other random selections of subject pairs, and also by using all of the 630 subject pairs, as shown in Figure 9. The results were very close to those in Figure 7. Therefore, the Dice coefficients are rather stable against random selections of subject pairs, and the results obtained based on a random selection of subject pairs can reflect those based on all subject pairs. Consequently, it is appropriate to choose one hundred pairs of subjects randomly in related calculations.

**Figure 10:**
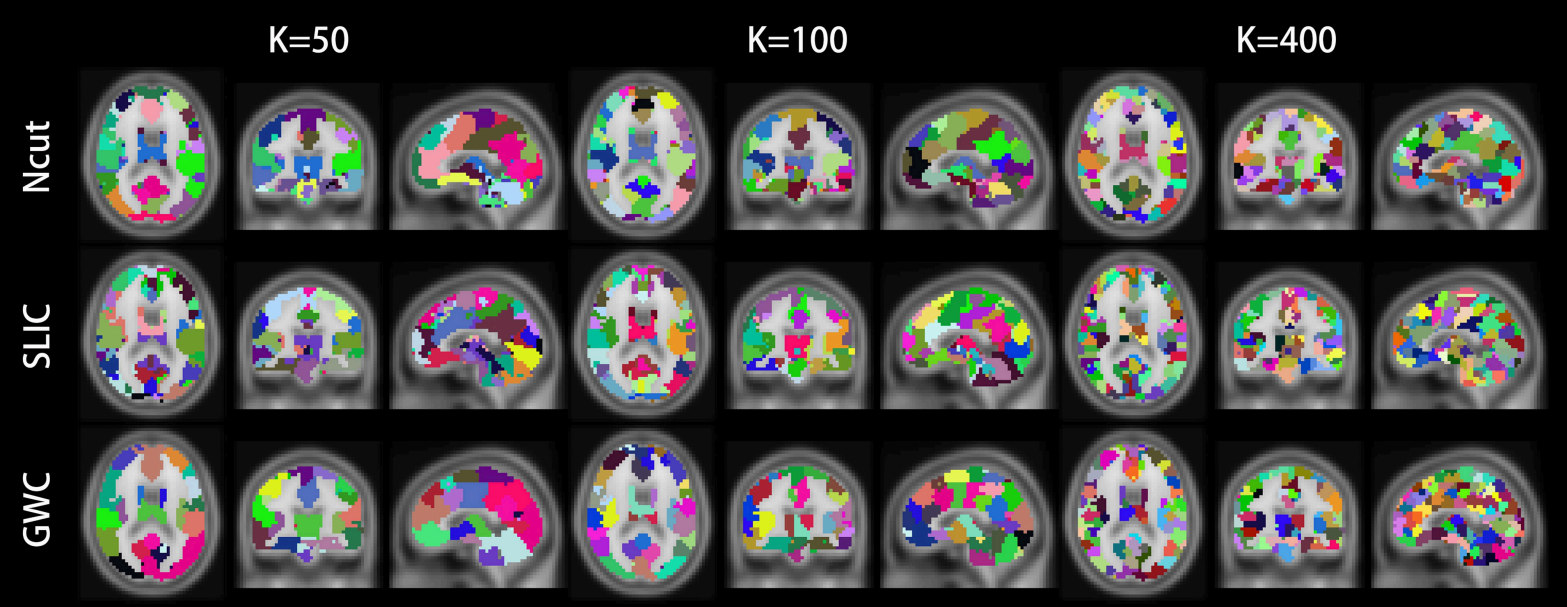
Illustrations of the atlases generated by the Ncut, SLIC, and GWC approaches when the brain of the first subject was parcellated into 50, 100, and 400 clusters. Each atlas is represented by its three orthogonal cross-sections. The colormap for each atlas is randomly generated, and each color represents a cluster.

**Figure 11:**
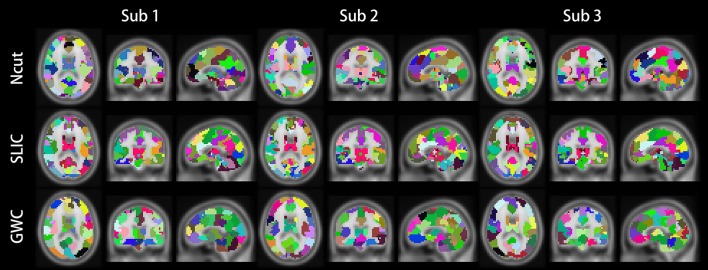
Illustrations of the atlases generated by the Ncut, SLIC, and GWC approaches when the brains of the first three subjects were parcellated into 100 clusters.

Figure 10 illustrates the atlases of the first subject when the brain is parcellated into 50, 100, and 400 clusters by the three approaches. Figure 11 illustrates the atlases of the first three subjects when the brains are parcellated into 100 clusters by the three approaches.

## Discussion

This study aims to improve the current RSFC-based parcellations. Specifically, its purpose is to parcellate the brain into spatially contiguous, functionally homogeneous, and reproducible clusters (Craddock et al., 2012). The major contributions of this study are threefold. First, we proposed a new subject-level whole-brain parcellation approach, i.e., combining supervoxels using the GWC approach. Clustering by combining supervoxels is better than clustering by aggregating voxels since supervoxel methods can effectively extract image structure, reduce image redundancy, provide a solid basis to compute local image features, and facilitate subsequent processing (Lucchi et al., 2012). Therefore, the GWC approach has a great methodological advantage over traditional parcellation approaches. Second, we ensured that the GWC approach does not rely heavily on spatial structures, thus avoiding the problem present in many previous parcellation approaches. Third, the GWC approach outperformed the two other approaches under different evaluation metrics, and therefore fulfilled our original purpose. The GWC approach would be useful in brain network analysis, especially when inter-subject variability is emphasized (Dubois and Adolphs, 2016). Some limitations of the GWC approach and potential ways for improvement are discussed below.

The GWC approach has many parameters that are difficult to tune. In this study, we chose the parameters empirically and changed one parameter at a time by fixing the other parameters in order to determine whether the parameters were appropriate. Since there is no standard criterion to evaluate a parcellation approach (Eickhoff et al., 2015; Wang and Wang, 2016), we cannot guarantee that our parameter settings are optimal. We can at most claim that GWC performs well under the metrics evaluated when certain parameters are used. It is also likely that the selected parameters are appropriate for the database used, but are not appropriate for another database. Therefore, when applying GWC to a different dataset, its parameters should be carefully tuned again. This is a common problem faced by parcellation approaches with tuning parameters.

To demonstrate that the GWC approach does not rely heavily on spatial structures, we compared the results obtained based on fMRI data to those obtained based on random data. If the results on fMRI data were close to the results on random data, we concluded that the parcellation approach relied heavily on spatial structures, and vice versa. However, whole-brain parcellation approaches almost inevitably rely on spatial structures since spatial structures are necessary to guarantee the spatial contiguity of the generated clusters (Wang and Wang, 2016). Ideally, comparisons among different parcellation approaches can be fair only when these approaches depend on spatial structures to a close degree. However, we have not figured out how to quantify this degree. It requires further investigation. The current study is only an initial attempt at judging whether a parcellation approach is reasonable at a very coarse level.

Many whole-brain RSFC-based parcellation studies are aiming to identify functionally connected networks (Salvador et al., 2005; van den Heuvel et al., 2008; Yeo et al., 2011). Among them, Salvador et al. (2005) applied hierarchical clustering to the mean time series generated from a 90-region anatomical template and defined six major systems, van den Heuvel et al. (2008) applied Ncut to perform whole-brain parcellation and revealed seven resting-state networks that showed large overlap with previously reported results, Yeo et al. (2011) applied a clustering algorithm based on von Mises-Fisher distribution to the cortical surface and resulted in 7- and 17-network cortical parcellations. The frequently reported networks including the default mode network, the attention network, and the auditory network were identified in these studies. However, the resultant networks in these studies may be too coarse to reveal some concealed brain connectome characteristics (de Reus and Van den Heuvel, 2013; Shen et al., 2013). The number of clusters in most of the latest studies is in the range of 50–500 (Fan et al., 2016; Glasser et al., 2016; Gordon et al., 2016; Arslan et al., 2017; Schaefer et al., 2017). Our study aims to generate clusters with fine granularities. Therefore, we set the cluster number to [25:25:500] in the experiments. The default mode network, the attention network, the auditory network, and other networks are thus being divided into smaller clusters.

The neurobiological meanings of the obtained parcellations are yet to be determined. This is usually assessed by visually comparing the generated brain atlases to task activations, myelin maps, cortical thickness, topography, electrical cortical stimulation maps, and the like (Blumensath et al., 2013; Laumann et al., 2015; Wang D. H. et al., 2015; Parisot et al., 2016; Arslan et al., 2017). However, there are several problems with such comparisons. First, they are based on the assumption that neuroimaging data with different modalities should yield similar parcellations, which has long been debated (Wig et al., 2011; Amunts et al., 2014; Eickhoff et al., 2015). Second, the transferability of the parcellations between different modalities is often far from perfect in practice (Glasser et al., 2016; Eickhoff et al., 2017). Third, it is difficult to quantify this comparison for parcellations with multiple granularities at the whole brain level. The situation becomes even more complex for individual subject-level parcellations considering the inter-subject variability. Therefore, we did not assess the neurobiological meanings of the obtained parcellations in this study.

Since GWC can conveniently incorporate multiple features, a potential way to improve the current parcellations is to use multi-modal neuroimaging data (Glasser et al., 2016) rather than resting-state fMRI data alone. This will naturally increase the neurobiological meaning of the generated brain atlases. Even when using resting-state fMRI data alone, we might explore other features such as 3D SIFT-based descriptors (Scovanner et al., 2007; Rister et al., 2017), 3D Ray descriptors (Lucchi et al., 2012), and 3D LBP with farer neighborhoods (Fehr and Burkhardt, 2008; Paulhac et al., 2008) in order to extract more information from the data. The necessity of these features requires further investigation. Additionally, while we constructed graphs for the average coordinates, features, and eigenvectors based on Euclidean distance, many other options can be tested. Recent studies have shown that sparse representation (Cheng et al., 2010) and low-rank representation (Liu et al., 2013) are suitable for graph construction due to several promising characteristics such as robustness to noise, sparsity, and data-adaptive neighborhood. These techniques might help to improve the parcellations.

It is very meaningful to compare the GWC approach to other parcellation approaches. In our study, GWC was compared to its random version to demonstrate that it does not rely heavily on spatial structures. In addition, GWC was compared to Ncut (Craddock et al., 2012; Shen et al., 2013) and SLIC (Wang and Wang, 2016; Wang et al., 2016) to demonstrate its advantages over other approaches. To include more parcellation approaches into comparison, there exists several problems since the configurations of different approaches are quite different. A few examples of such differences are described below: Gallardo et al. (2017) focused on structural parcellation rather than functional parcellation; Ryali et al. (2013) focused on parcellating a ROI rather than the whole brain; Honnorat et al. (2015), Parisot et al. (2016), and Gordon et al. (2016) focused on group-level parcellations rather than individual subject-level parcellations; Gordon et al. (2016), Fan et al. (2016), and Glasser et al. (2016) parcellated the brain into a fixed resolution rather than multiple resolutions. Additionally, a large number of parcellation studies including those by Gordon et al. (2016), Glasser et al. (2016), and Schaefer et al. (2017) performed parcellation in surface space rather than volume space. As a special case, the study by Arslan et al. (2017) is highly relevant to the current study, although it focused on surface-based analysis. In Arslan et al. (2017), ten subject-level whole-brain parcellation methods including Ncut, K-means, hierarchical clustering, geometric clustering, and random clustering were evaluated systematically. In our study, both Ncut and random clustering were included for comparison. The Ncut approach is free from strong assumptions on data distribution, robust to outliers and random initializations, easy to implement, and reasonably fast for huge graphs (Shi and Malik, 2000; Yu and Shi, 2003; von Luxburg, 2007). Therefore, it has great advantages over traditional clustering approaches (Ng et al., 2002). Moreover, the Ncut approach has been successfully applied to brain parcellation and outperforms the competing approaches in this area (van den Heuvel et al., 2008; Shen et al., 2010, 2013; Craddock et al., 2012). The SLIC approach is a novel whole-brain parcellation approach that has been demonstrated to outperform Ncut in different situations (Wang and Wang, 2016; Wang et al., 2016). With the above considerations, we chose the Ncut and SLIC approaches as competing approaches and did not consider other approaches.

It might be possible to modify the latest parcellation approaches (Gordon et al., 2016; Parisot et al., 2016; Gallardo et al., 2017; Schaefer et al., 2017) in several major aspects in order to make a direct comparison between these approaches and the proposed GWC approach. First, they should be RSFC-based approaches. Second, the parcellations should be performed in the whole brain. Third, the parcellations should be performed at the individual subject level. Fourth, the numbers of clusters should be varied in the same range. Fifth, it is better to restrict all competing approaches in one space, no matter whether it is volume space or surface space, because a parcellation optimized in one space may not perform well in another space. Sixth, the parcellation approaches should depend on spatial structures to a close degree. Only when these requirements are satisfied can we make a fair and thorough comparison between the modified approaches and the GWC approach. However, the approaches in most parcellation studies are highly specialized. The potential to extend them to meet our requirements might thus be limited. This area also requires further investigation.

Despite the aforementioned limitations, the proposed GWC approach successfully fulfills the original purpose of RSFC-based parcellations, i.e., parcellating the brain into spatially contiguous, functionally homogeneous, and reproducible clusters. Therefore, it can be utilized to construct reliable functional networks for brain network analysis. Previously, RSFC-based parcellations have been successfully used to track ongoing cognition (Gonzalez-Castillo et al., 2015), to identify individuals (Finn et al., 2015), to measure sustained attentional abilities (Rosenberg et al., 2016), and to predict age (Liem et al., 2017). In a recent literature review, Hallquist and Hillary (2018) reported that more than 50 distinct parcellation techniques have been used to investigate brain disorders in 106 studies. The clinical disorders presented in these studies included Alzheimer's disease, epilepsy, depression, schizophrenia, etc. The GWC approach offers an alternative and possibly a better choice in similar applications. Therefore, we remain optimistic about this approach and expect it to facilitate related studies.

## Conclusion

This study aimed to improve RSFC-based parcellation approaches in order to construct more reliable brain networks. In this study, we introduced a new supervoxel-based approach, i.e., the GWC approach, to perform whole-brain parcellation for individuals. The parameters of GWC were selected empirically, and we showed that our choices were generally appropriate by tuning one parameter at a time. By comparing the results of the GWC approach to those of the random GWC approach, we demonstrated that GWC does not rely heavily on spatial structures. This reflects a great advantage over many previous whole-brain parcellation approaches. By comparing the GWC approach to the modified Ncut and SLIC approaches, we found that GWC outperformed Ncut and SLIC in terms of spatial contiguity and reproducibility, and led to comparable results in terms of functional homogeneity. Therefore, the performance of GWC is satisfying. As an improved RSFC-based parcellation approach, GWC might have applications in various studies related to brain network analysis, e.g., cognition, development, aging, disease, and personalized medicine. Since GWC could conveniently incorporate multiple features, it has the potential to integrate multi-modal neuroimaging data and thus naturally increase the neurobiological meanings of the generated brain atlases. Additionally, we might extract other types of features from brain data to improve the GWC approach.

## Author contributions

JW and HW: Designed the study; JW: Analyzed the data and drafted the manuscript under the supervision of HW; ZH and HW: Revised the manuscript. All authors approved the final version of the manuscript.

### Conflict of interest statement

The authors declare that the research was conducted in the absence of any commercial or financial relationships that could be construed as a potential conflict of interest. The reviewer [GG] and handling Editor declared their shared affiliation, and the handling Editor states that the process nevertheless met the standards of a fair and objective review.
