# Supplementary material for: Generation of Individual Whole-Brain Atlases With Resting-State fMRI Data Using Simultaneous Graph Computation and Parcellation
Source: Front Hum Neurosci. 2018 May 4;12:166. doi: 10.3389/fnhum.2018.00166 (PMC5945868; doi:10.3389/fnhum.2018.00166)
Supplement: Supplementary file 1 [file Data_Sheet_1.PDF]

Supplementary Material

Generation of individual whole-brain atlases with resting-state fMRI data using simultaneous graph computation and parcellation

J. Wang[1,2], Z. Hao[1], H. Wang[1*]

[1]School of Mathematics and Big Data, Foshan University, Foshan, 528000, Guangdong, China

[2]Key Laboratory of Child Development and Learning Science of Ministry of Education, Research Center for Learning Science, Southeast University, Nanjing, 210096, Jiangsu, China

*** Correspondence:**
Dr. Haixian Wang
26187954@qq.com

1 Quadratic programming I

Consider the following quadratic programming problem

$$\min_{x} \frac{1}{2}\|x-v\|_2^2 \quad s.t.\ x^T\mathbf{1}=1, x\geq 0, \tag{1}$$

where $v \in R^n$ is a constant vector, $x \in R^n$ is the target variable. The Lagrangian function of problem (1) is

$$\mathcal{L}(x,\eta,\theta)=\frac{1}{2}\|x-v\|_2^2-\eta(x^T\mathbf{1}-1)-\theta^T x, \tag{2}$$

where $\eta \in R$, $\theta \in R^n$ are the Lagrange multipliers. According to the KKT condition (Boyd and Vandenberghe, 2004), we have

$$x^T\mathbf{1}=1, \tag{3}$$

$$x\geq 0, \tag{4}$$

$$\theta\geq 0, \tag{5}$$

$$\theta_i x_i=0, i=1,2,\dots,n, \tag{6}$$

$$x = v + \eta + \theta. \tag{7}$$

According to (6) and (7), we have

$$\theta_i x_i = (x_i - v_i - \theta)x_i = 0, i = 1,2, \dots, n. \tag{8}$$

When $v_i + \eta > 0$, according to (5) and (7), we have $\theta_i = x_i - v_i - \eta \geq 0$, and therefore $x_i \geq v_i + \eta > 0$. (8) holds only when $x_i - v_i - \eta = 0$, or equally $x_i = v_i + \eta$. When $v_i + \eta \leq 0$, if $x_i > 0$, then $\theta_i x_i = (x_i - v_i - \eta)x_i > 0$, which violates (8); if $x_i = 0$, (8) holds; if $x_i < 0$, this condition violates (4). To summarize, we have $x_i = (v_i + \eta)_+$, $i = 1,2, \dots, n$, or equally

$$x = (v + \eta)_+. \tag{9}$$

According to (3) and (9), we have

$$(v + \eta)_+^T \mathbf{1} = 1. \tag{10}$$

Since the left side is a piecewise-linear function of η, problem (10) has a unique solution. To solve η, suppose that the number of nonzero elements in x is k ($1 \leq k < n$), u is a vector formed by sorting the elements of v in descending order, and then we have

$$u_1 + \eta \geq u_2 + \eta \geq \cdots \geq u_k + \eta > 0 \geq u_{k+1} + \eta \geq \cdots \geq u_n + \eta. \tag{11}$$

It requires that

$$\eta \in (-u_k, -u_{k+1}]. \tag{12}$$

According to (10) and (11), we have

$$x^T \mathbf{1} = \sum_{i=1}^{k} (u_i + \eta) = 1. \tag{13}$$

By solving η in (13), we have

$$\eta = \frac{1}{k} - \frac{1}{k} \sum_{i=1}^{k} u_i \,. \tag{14}$$

According to (12) and (14), k satisfies the following condition

$$\frac{1}{k}-\frac{1}{k}\sum_{i=1}^{k}u_i \in (-u_k, -u_{k+1}]. \tag{15}$$

This condition determines the unique k value. Then we could obtain η and x according to (14) and (9) respectively. Or equivalently, we could alternatively iterate η and k according to (11) and (14) until convergence to solve x. A special case that requires additional attention is that when the elements of v are the same, the above discussions become invalid. In that case, the optimal solution is $x = 1/n$. Since that case seldom happens, it can be neglected in practice. The code for problem (1) could be found at https://github.com/yuzhounh/GWC/blob/master/quadprog_can.m.

2 Quadratic programming II

Consider the following quadratic programming problem

$$\begin{aligned} &\min_{x} \frac{1}{2}\left\|x-\frac{v}{\alpha}\right\|_2^2 \\ &s.t.\ x^T\mathbf{1}=1, x \geq 0, \end{aligned} \tag{16}$$

where $v \in R^n$ is a constant vector, $x \in R^n$ is the target variable, $\alpha \in R$ is a tuning parameter that adjusts the number of nonzero elements (k) in x. In some practical problems, since k is an integer, it is more convenient to tune k than to tune α. Therefore, we fix the k value to determine α and solve x for problem (16). The Lagrangian function of problem (16) is

$$\mathcal{L}(x,\eta,\theta)=\frac{1}{2}\left\|x-\frac{v}{\alpha}\right\|_2^2-\eta(x^T1-1)-\theta^T x, \tag{17}$$

where $\eta \in R$ and $\theta \in R^n$ are the Lagrange multipliers. According to (9), the solution of problem (16) is

$$x=\left(\frac{v}{\alpha}+\eta\right)_+. \tag{18}$$

Suppose u is a vector formed by sorting the elements of v in descending order, according to (14) and (15), we have

$$\eta = \frac{1}{k} - \frac{1}{k}\sum_{i=1}^{k}\frac{u_i}{\alpha} \in \left(-\frac{u_k}{\alpha}, -\frac{u_{k+1}}{\alpha}\right]. \tag{19}$$

Let

$$\frac{1}{k} - \frac{1}{k}\sum_{i=1}^{k}\frac{u_i}{\alpha} = -\frac{u_{k+1}}{\alpha}. \tag{20}$$

Then we have

$$\alpha = \sum_{i=1}^{k} u_i - k u_{k+1}. \tag{21}$$

Then we could obtain η and x according to (19) and (18) respectively. Therefore, when the number of nonzero elements (k) of x is given, we could determine the parameter α and obtain the explicit solution of x to problem (16). The code for problem (16) could be found at: https://github.com/yuzhounh/GWC/blob/master/quadprog_can_explicit.m.

3 Tuning the parameter m in SLIC and random SLIC

It is worthwhile to set the parameter m to other values in order to check how the parcellation performances of the SLIC and random SLIC approaches are affected by this parameter.

When m is small enough, the unified distance, i.e., equation (24) in the main text, approximates the functional distance multiplying a ratio, i.e.,

$$d_{ij} \approx \frac{\left\|v_i - v_j\right\|_2}{m}.$$

The ratio will not affect the clustering results because it is the comparison between two distances rather than the distance itself determines the label of a voxel. Therefore, when m is small enough, the unified distance can be equivalently written as

$$d_{ij} = \left\|v_i - v_j\right\|_2.$$

That is, the unified distance equals the functional distance. We denote this extreme condition as $m = 0$.

In another extreme condition, i.e., when m is large enough, we have

$$d_{ij} \approx \frac{\|u_i - u_j\|_2}{S},$$

or equivalently

$$d_{ij} = \|u_i - u_j\|_2$$

for the same reason. That is, the unified distance equals the spatial distance. We denote this extreme condition as $m = Inf$. In this case, since the unified distance does not include functional distance, the parcellation is no longer determined by fMRI data or random fMRI data. Therefore, the SLIC and random SLIC approaches reduce to the same approach, and the clustering results are the same for different subjects.

Except for the above two extreme conditions, we also tested the SLIC and random SLIC approaches with m =10, 20, 30, and 40.

The results of the SLIC and random SLIC approaches with the above six m values are shown in Supplementary Figures 1-6. To reduce computational time, the experiments were conducted only on three subjects and the number of clusters was sampled to [50:50:1000]. Since the initialized cluster number was set to [25:25:500] in the main text, we just focus our attention on the results obtained when the cluster numbers were no larger than 500. The remaining results are provided for references. The results in Supplementary Figures 2 and 5 were generally consistent with the corresponding results in Figure 6 in the main text.

Then we compared the results across different m values. When $m = 0$, the performances of the SLIC and random SLIC approaches were rather bad, especially in terms of spatial contiguity and reproducibility. When m was larger than 10, the differences of performances between SLIC and random SLIC diminished with increasing m value. The results indicate that SLIC with m larger than 10 relies heavily on spatial structures. In conclusion, setting m to 10 is a reasonable choice for the SLIC approach.

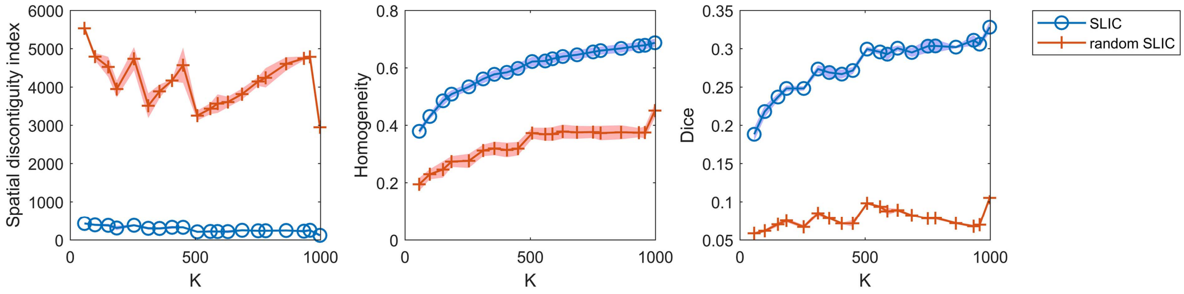

Supplementary Figure 1. The results of different evaluation metrics for the SLIC and random SLIC approaches with $m = 0$.

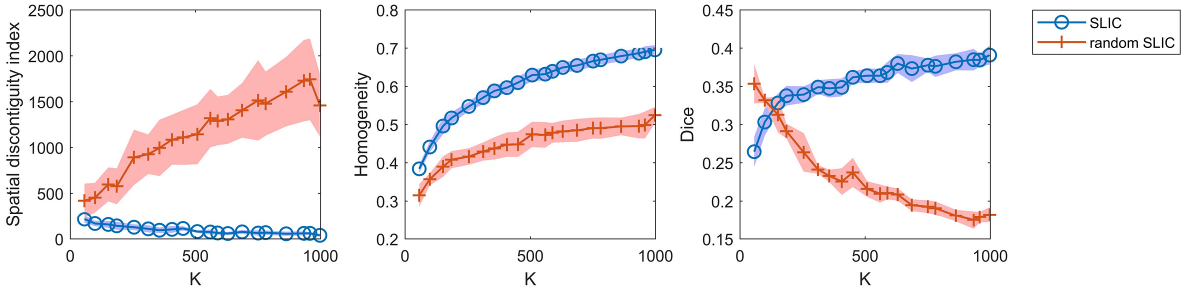

Supplementary Figure 2. The results of different evaluation metrics for the SLIC and random SLIC approaches with $m = 10$.

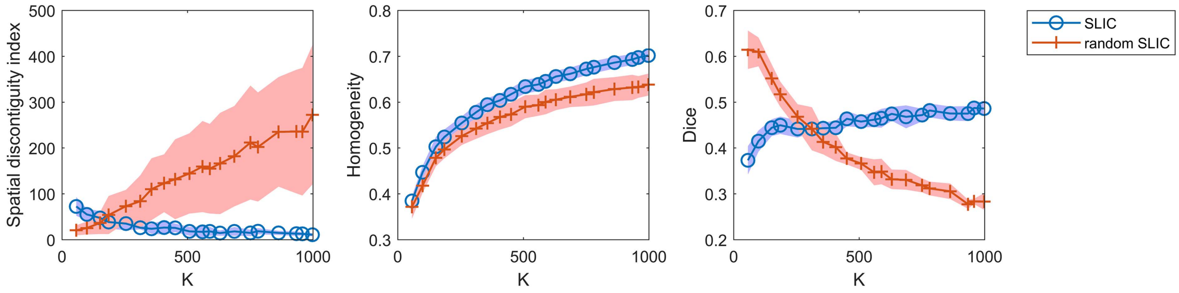

Supplementary Figure 3. The results of different evaluation metrics for the SLIC and random SLIC approaches with $m = 20$.

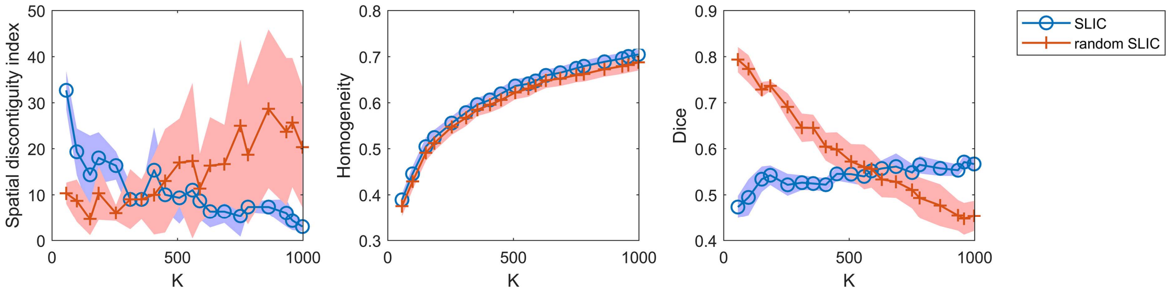

Supplementary Figure 4. The results of different evaluation metrics for the SLIC and random SLIC approaches with $m = 30$.

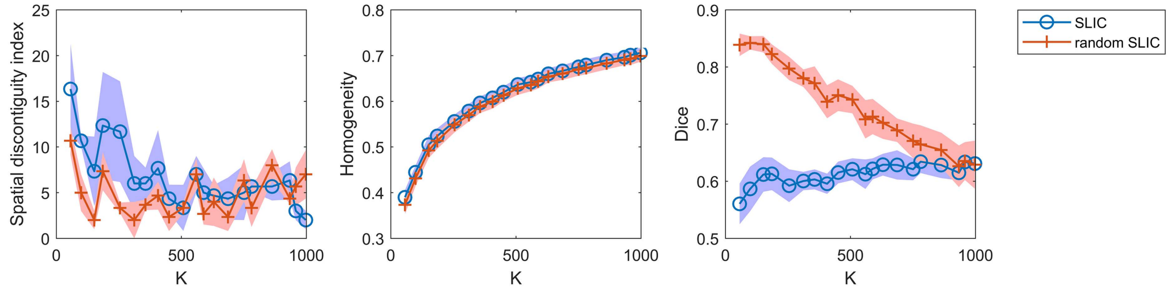

Supplementary Figure 5. The results of different evaluation metrics for the SLIC and random SLIC approaches with $m = 40$.

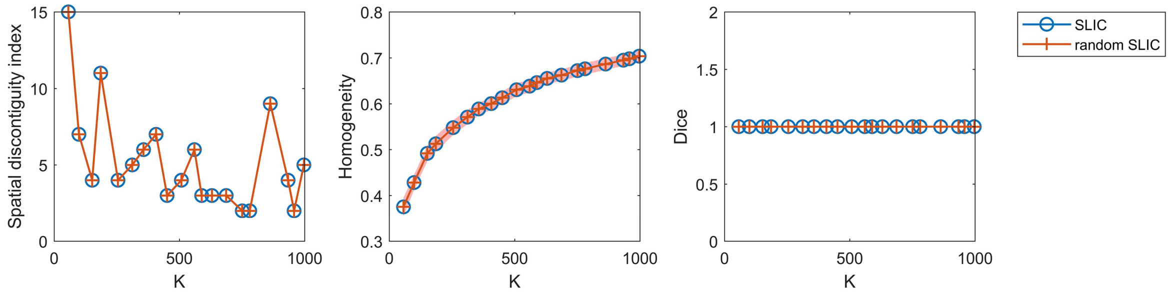

Supplementary Figure 6. The results of different evaluation metrics for the SLIC and random SLIC approaches with $m = Inf$.

4 Comparison of the three approaches

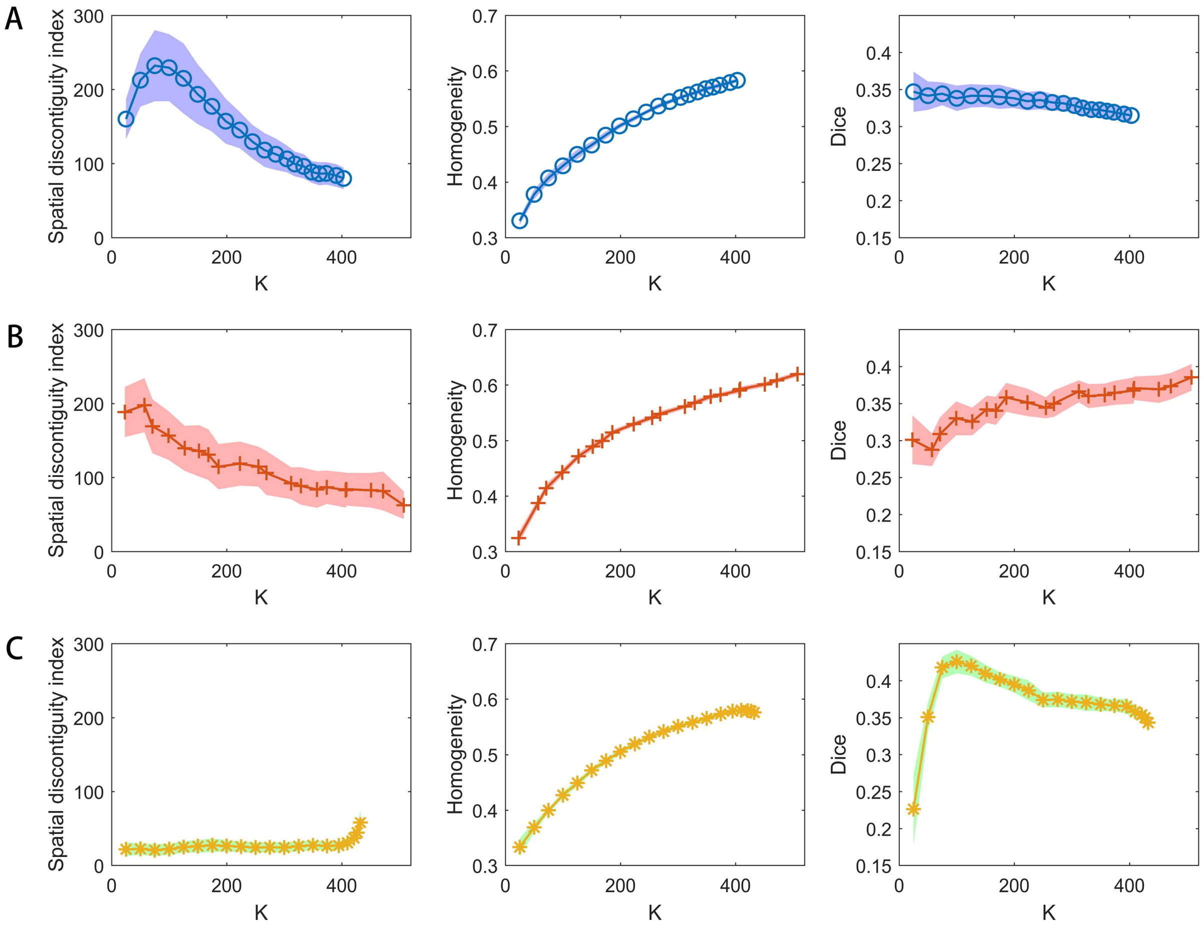

Supplementary Figure 7. The results of different evaluation metrics for the (A) Ncut, (B) SLIC, and (C) GWC approaches. This is an alternative to Figure 7 in the main text.

References

Boyd, S., and Vandenberghe, L. (2004). *Convex optimization.* Cambridge university press.
